# From Mechanism to Clinic: Engineered Bacteria–Nanomaterial Hybrid Systems for Cancer Immunotherapy

**DOI:** 10.34133/research.1390

**Published:** 2026-08-11

**Authors:** Jia Lin, Mingxin An, Yuxin Dai, Yuanting Huang, Zhijun Li, Pin Yao, Huizhe Wu, Dake Xu, Xiaoqian Xu

**Affiliations:** ^1^ School of Materials Science and Engineering, Northeastern University, Shenyang 110819, China.; ^2^ School of Intelligent Medicine, China Medical University, Shenyang 110122, China.; ^3^ Key Laboratory of Cell Biology, Ministry of Public Health and Key Laboratory of Medical Cell Biology, Ministry of Education, China Medical University, Shenyang 110122, China.; ^4^ National Clinical Research Center for Women’s Health and Obstetric and Gynecologic Diseases, Department of Obstetrics and Gynecology, Peking Union Medical College Hospital, Chinese Academy of Medical Sciences & Peking Union Medical College, Beijing 100005, China.; ^5^ Department of Health Management, Shenyang Women’s and Children’s Hospital, Shenyang, Liaoning 110011, China.; ^6^ Department of Pharmacology, Scientific Experimental Center, School of Pharmacy, China Medical University, Shenyang 110122, China.

## Abstract

Hypoxia, immunosuppression, and pronounced heterogeneity within the tumor microenvironment (TME) hinder the effectiveness of cancer therapies. Engineered bacteria–nanomaterial hybrid systems have emerged as a promising approach to address these challenges. Bacterial chassis provide active tumor targeting, deep tissue penetration, and in situ proliferation, facilitating the precise delivery of immunomodulators. Concurrently, nanomaterials interfaced with these living carriers can be activated by external physical stimuli, inducing photothermal, photodynamic, sonodynamic, and magnetothermal effects within solid tumors. These interactions promote immunogenic cell death (ICD) and enable real-time monitoring. Recent advances in synthetic biology and nanotechnology have led to the development of an expanding range of preclinical biohybrid platforms, while several related components, including bacterial therapeutics, bacterial derivatives, and physically activated nanomedicine platforms, have progressed into clinical evaluation. This review first explores the origins and roles of tumor-associated bacteria. It then summarizes strategies for engineering bacteria–nanomaterial hybrid systems. Subsequently, this review examines how physical stimuli enhance targeting, remodel the TME, and amplify antitumor immunity. Finally, safety, manufacturing, and regulatory challenges impacting clinical translation are discussed. Overall, these platforms offer a potentially powerful framework for precision cancer immunotherapy. However, successful clinical translation will require stronger evidence regarding safety, controllability, manufacturing consistency, and therapeutic efficacy.

## Introduction

Malignant tumors remain a leading cause of global mortality, with their biological complexity and heterogeneity presenting substantial challenges to effective treatment [1]. While conventional therapies such as chemotherapy have extended survival, their lack of tumor specificity often leads to suboptimal intratumoral drug concentrations and dose-limiting systemic toxicities from off-target effects [2]. Although immune checkpoint inhibitors have improved outcomes in a subset of patients, resistance—whether primary or acquired, including immune tolerance—remains prevalent [3]. Furthermore, the tumor microenvironment (TME) is often characterized by severe hypoxia and immunosuppression, further reducing therapeutic efficacy [4]. Consequently, oncology is increasingly shifting from surgery, chemotherapy, and radiotherapy toward novel strategies, including immunotherapy, molecularly targeted agents, and bacteria-based therapies, to overcome these persistent challenges [5]. Accordingly, there is a clear need for strategies that can access hypoxic niches, deliver therapeutic payloads with high precision, enable spatiotemporally controllable activation, and convert localized tumor destruction into systemic antitumor immunity.

Bacteria-based therapies have emerged as a promising area of research in oncology [6]. Some bacteria display intrinsic chemotaxis and immunostimulatory properties, allowing selective accumulation within tumors and the induction of antitumor immunity, which partially mitigates the limitations of traditional therapies [7]. Anaerobic and facultative anaerobic bacteria, in particular, preferentially colonize hypoxic and necrotic regions of solid tumors, utilizing local nutrients to persist and suppress tumor growth through nutrient competition and inflammation-driven mechanisms [8]. These properties suggest that bacteria can not only antagonize tumors directly but also serve as natural vectors for targeted drug and therapeutic material delivery. Engineered bacteria act as living therapeutics, expressing and locally releasing cytotoxic effectors—such as toxins, antigens, and enzymes—that directly kill cancer cells and enhance host antitumor immunity [9]. Tumor-targeting strains, including the probiotic *Escherichia coli Nissle* 1917 (EcN), attenuated *Salmonella*, *Bifidobacterium*, and *Listeria*, exhibit preferential tumor accumulation and potent immune stimulation [10]. However, clinical translation remains hindered by residual virulence, potential pathogenicity, treatment safety and stability concerns, and the need for precise control over cargo expression and release [11], prompting the integration of engineered bacteria with complementary therapeutic strategies to reduce risks and enhance overall efficacy.

Simultaneously, the emergence of nanotechnology has provided oncology with powerful tools. Due to their nanoscale size, tunable surfaces, and controllable release profiles, nanomaterials are increasingly used in cancer diagnosis and therapy [12]. By enabling high-capacity cargo loading and protection, tunable surface engineering, and programmable release, nanomaterials can improve pharmacokinetics and enhance tumor-localized delivery relative to free drugs [13]. In immuno-oncology, custom-designed nanoplatforms allow precise targeting of immune cell subsets and active modulation of the tumor immune microenvironment, reducing immune-related adverse effects and optimizing antitumor responses [14]. Importantly, stimulus-responsive nanomaterials, activated by endogenous cues or exogenous physical triggers, can induce localized cytotoxicity and promote ICD, thereby linking focal tumor damage with downstream immune activation [15]. Despite its potential, the efficacy of nanomedicine is limited by the structural characteristics of the TME. The dense extracellular matrix (ECM), abnormal vasculature, hypoxic conditions, and elevated interstitial fluid pressure together hinder the uniform penetration of therapeutic agents within tumors. Consequently, nanoparticles (NPs) relying solely on passive accumulation through the enhanced permeability and retention (EPR) effect often exhibit shallow and uneven distribution, leaving residual disease in heterogeneous solid tumors. Single-modality nanotherapy is insufficient to address the complexity and cellular diversity of the TME. Enhancing antitumor efficacy will require next-generation platforms capable of actively targeting and penetrating deeper tissues, utilizing stimuli-responsive mechanisms, externally triggered transport, or other strategies to overcome stromal and perfusion barriers.

**Fig. 1.**
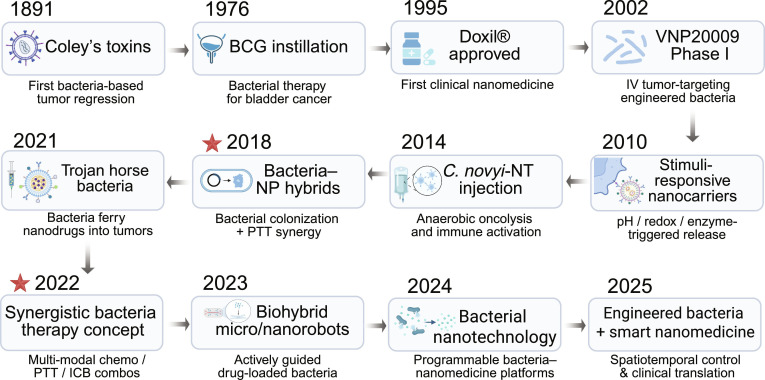
Technological progress of engineered bacteria–nanomaterial hybrid systems in cancer treatment. ICB, immune checkpoint blockade.

To overcome these challenges, engineered bacteria–nanomaterial hybrid systems have emerged as a promising approach in cancer immunotherapy [16]. Advancements in synthetic biology now enable the genetic reprogramming of bacteria to reduce virulence while enhancing intratumoral colonization and the delivery of therapeutic payloads, thus improving both safety and efficacy [17]. In 2018, bacteria–NP hybrids harnessed bacterial colonization to concentrate photothermal nanodrugs in tumors, providing a blueprint for using motile microbes as carriers for stimulus-responsive nanomedicine. In 2022, the “synergistic bacteria therapy” concept extended this paradigm to multimodal chemo–photothermal–immune checkpoint combinations, defining engineered bacteria–nanomaterial hybrid systems as integrated, programmable systems for precision cancer immunotherapy (Fig. 1). This strategy combines synthetic biology with nanobiotechnology to create biohybrids that enable multimodal, programmable synergy: Nanomaterials enhance bacteria-mediated drug delivery and antitumor efficacy, while the bacteria’s ability to infiltrate deep into tumors and remodel the TME accelerates the localized action of nanomaterials [18]. Additionally, nanomaterials can be tethered to, coated onto, internalized by, or biomineralized within bacterial chassis, thereby increasing payload capacity and improving control over release kinetics [19]. These features allow bacteria–nanomaterial biohybrid platforms to combine active tumor targeting with stimulus-responsive therapeutic functions, supporting targeted delivery, TME remodeling, and antitumor immune activation.

**Fig. 2.**
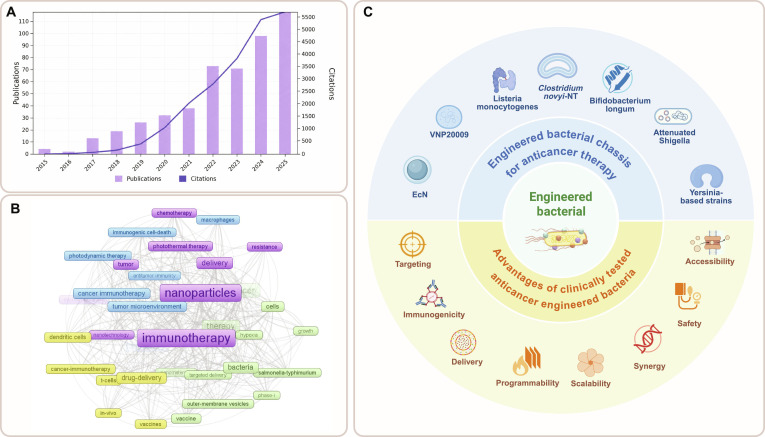
Bibliometric networks of global research on engineered bacteria–nanomaterial hybrid systems in cancer immunotherapy. (A) Annual publications and citations in this field from 2015 to 2025. (B) Keyword co-occurrence network of research topics. (C) Engineered bacterial chassis and selection criteria for antitumor therapy.

Bibliometric analysis indicates that this field began to emerge around 2015 and expanded rapidly after 2017, with research themes converging on immunotherapy, drug delivery, and tumor microenvironment modulation (Fig. 2). Accordingly, this review focuses on hybridization strategies, dual-responsive activation, immune remodeling, and translational barriers that determine the clinical feasibility of these systems.

These hybrids are best viewed as a single therapeutic architecture in which the bacterial and material modules perform distinct but interdependent roles. The bacterial chassis enables tumor tropism, colonization of hypoxic or immunosuppressed niches, programmable cargo production, and pathogen-associated molecular pattern (PAMP)-driven immune priming. The nanomaterial module adds functions that bacteria alone usually cannot provide, including imaging, activation by external fields or tumor-derived cues, focal tumor cell injury, and amplification of ICD-associated immune responses. Their value therefore lies in forming programmable living systems that integrate 4 linked functions: bacterial tumor navigation and colonization, programmable therapeutic output, externally or microenvironmentally triggered nanomaterial activation, and subsequent immune amplification. Accordingly, the following sections are organized around an engineering workflow: chassis selection, genetic programming, interface construction, payload loading, stimulus response, controlled release, immune output, safety control, and manufacturing considerations.

In this review, a bona fide live engineered bacteria–nanomaterial hybrid is defined by 3 minimum criteria: First, the system retains a live engineered bacterial chassis as the tumor-homing and/or programmable component; second, the nanomaterial module is physically, chemically, or biologically associated with the bacteria in a stable and functionally relevant manner; and third, the therapeutic or imaging effect requires this coupling rather than mere co-administration. Systems based solely on engineered bacteria, nanomaterials, bacterial derivatives such as outer membrane vesicles (OMVs), bacteria-inspired materials, or separately administered bacteria–NP combinations are discussed as related or supporting technologies, but are not classified here as bona fide live hybrids.

## The Role of Bacteria in Tumor Initiation and Progression

Bacteria play an important role in oncogenesis and cancer progression. It is estimated that 15% to 20% of malignancies worldwide can be directly attributed to microbial infections [20]. Bacteria and other microbes can reside within the TME and, through various mechanisms, influence tumor initiation, progression, and therapeutic responses [21]. This section systematically examines these roles, discussing the sources of tumor-associated bacteria, the mechanisms of bacteria–tumor crosstalk, and the criteria for selecting bacterial strains as chassis for therapeutic engineering.

### Sources and colonization of bacteria in tumor tissues

**Fig. 3.**
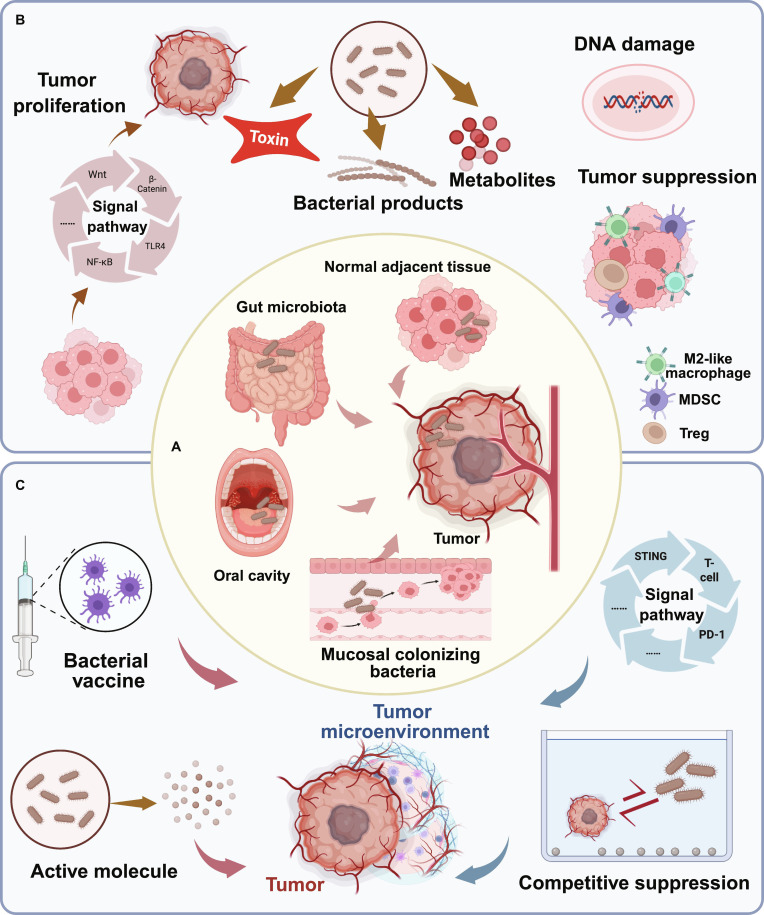
Interactions between tumors and bacteria in vivo, showing bacteria-mediated tumor-promoting and tumor-suppressive mechanisms. (A) Major sources of intratumoral bacteria. (B) Role of the microbiota in promoting tumors. (C) Role of the microbiota in inhibiting tumors.

The origin of intratumoral bacteria is multifactorial (Fig. 3A). First, mucosal microbiota at the organ of tumor origin can translocate across damaged epithelium into the lesion; thus, tumors arising in mucosal organs—such as colorectal, lung, cervical, and pancreatic cancers—frequently harbor bacteria from the corresponding mucosal niches [22]. Second, microbial communities in peritumoral normal tissues often resemble those within the tumor, suggesting migration and subsequent colonization from adjacent sites. Third, bacteria originating from distant niches, such as the oral cavity or the gut, can reach tumors through the bloodstream or lymphatic system [23]. A notable example is the oral commensal *Fusobacterium nucleatum*, which can disseminate through the bloodstream and colonize tumors, establishing intralesional colonization and completing oral-to-tumor migration [24]. Collectively, tumor-associated bacteria are closely connected to the host’s microbiota, with the oral and intestinal communities representing major sources of intratumoral microbes.

However, not all exogenous or commensal bacteria that enter the body can establish residency within tumors. Successful colonization is facilitated by specific features of the TME—including immunosuppression, hypoxia, and nutrient enrichment—which create niches conducive to bacterial survival and growth [25]. Additionally, aberrant tumor vasculature and compromised barrier integrity provide entry points: Endogenous microbes may breach damaged mucosa, spread from adjacent tissues, or disseminate hematogenously or lymphatically, ultimately colonizing regions of weaker immune surveillance within the tumor.

### Mechanisms of bacteria–tumor interactions

Once inside the tumor, bacteria interact with both malignant and stromal compartments through various context-dependent mechanisms. These interactions can be dual in nature: They can promote tumor initiation, progression, and therapeutic resistance, but under appropriate conditions, they can also elicit direct cytotoxicity and enhance antitumor immunity.

#### Mechanisms by which bacteria promote tumorigenesis

Certain tumor-associated bacteria produce genotoxins that directly damage host DNA, contributing to mutagenesis and genomic instability (Fig. 3B). In colorectal tumors, *E. coli* strains harboring the *pks* genomic island synthesize colibactin, which induces DNA double-strand breaks and related lesions in host cells, thereby promoting neoplastic transformation of colonic epithelium [26]. Similarly, *Helicobacter pylori* secretes the virulence factor CagA, which activates epithelial β-catenin signaling, driving abnormal proliferation and gastric mucosal carcinogenesis [27]. In addition to direct genotoxic effects, chronic bacterial infections sustain inflammation, leading to oxidative and nitrosative stress that damages DNA and disrupts repair mechanisms, ultimately increasing the mutational burden. These processes position bacteria as potential “initiators” in tumorigenesis, destabilizing the host genome at early stages.

Beyond direct genotoxicity, intratumoral bacteria can reprogram host signaling through structural components and metabolites, promoting malignant behaviors such as proliferation and invasion. Bacterial components, such as lipopolysaccharides (LPSs), activate Toll-like receptor 4/nuclear factor-κB (TLR4/NF-κB)-driven inflammatory cascades, inducing pro-tumorigenic cytokines and chemokines that create a growth-permissive environment [28]. Microbial metabolites also reshape cellular and microenvironmental metabolism. Gut-derived secondary bile acids, such as deoxycholic acid, drive cancer-associated fibroblasts toward pro-tumor phenotypes. Lithocholic acid further modulates T helper 17 (Th17)/regulatory T cell (Treg) differentiation, facilitating immune evasion [29]. Additionally, certain bacteria detected in lung tumors can synthesize methionine, providing methyl donors that enhance DNA methylation in cancer cells, thereby promoting epigenetic progression [23]. Collectively, bacterial toxins, enzymes, and metabolites converge to modulate host signaling and metabolic pathways, sculpting a pro-carcinogenic TME.

Certain intratumoral bacteria can also suppress antitumor immunosurveillance, thereby promoting cancer cell survival and dissemination. Chronic bacteria-driven inflammation is often associated with immunosuppressive phenotypes, such as the polarization of macrophages toward the M2 state and the recruitment of Tregs and myeloid-derived suppressor cells (MDSCs), which dampen effector immune responses [30]. Dysbiosis in pancreatic cancer tissue has been shown to suppress both innate and adaptive immunity, accelerating tumorigenesis [31]. Moreover, bacteria can directly impair lymphocyte function via specific molecular interactions. For example, *F. nucleatum*, upon colonizing colorectal tumors, engages inhibitory receptors such as TIGIT and CEACAM1 on tumor-infiltrating lymphocytes, reducing natural killer (NK) and T cell cytotoxicity and promoting immune escape [32]. Additionally, *F. nucleatum* infection down-regulates the m^6^A methyltransferase METTL3 in colorectal cancer cells, enhancing stemness and metastatic potential [33,34]. In summary, by fostering an immunosuppressive microenvironment and co-opting immune checkpoint pathways, bacteria create conditions conducive to tumor cell evasion, invasion, and metastasis.

#### Mechanisms of bacteria-mediated antitumor activity

As potent immune stimuli, bacteria can mobilize both innate and adaptive immune responses, thereby exerting indirect tumor-suppressive effects (Fig. 3C). Historically, William Coley’s observations that streptococcal infections occasionally preceded spontaneous tumor regressions inspired the development of mixed bacterial vaccines for cancer therapy [23]. Clinically, the attenuated live vaccine Bacille Calmette–Guérin (BCG) has been successfully used in bladder cancer, where it induces robust local immune responses that eliminate tumor cells [35]. Beyond exogenous vaccination, specific intratumoral or peritumoral microbes can enhance host antitumor immunity. For example, gut-derived Bifidobacterium can migrate to and accumulate within tumors, activating innate immune pathways such as the stimulator of interferon genes (STING) pathway, thereby enhancing T cell-mediated antitumor responses. In murine models, Bifidobacterium administration markedly improves responses to programmed cell death protein 1 (PD-1) checkpoint blockade [36]. In prostate cancer, certain intratumoral commensals, such as *Pseudomonas spp*. and *E. coli*, correlate with reduced metastasis and increased infiltration of effector CD8^+^ T cells. These findings suggest that such bacteria may slow disease progression by augmenting tumor immunogenicity [37].

Beyond immune modulation, intratumoral bacteria can compete metabolically with cancer cells. Proliferating microbes consume local nutrients and oxygen, and in the resource-limited TME, this competition can deprive malignant cells of essential substrates, thereby slowing tumor growth. Additionally, several bacterial species produce molecules with direct antitumor effects: Probiotic strains that generate short-chain fatty acids can suppress cancer cell proliferation and induce differentiation or apoptosis; clostridial species such as *Clostridium perfringens*, which proliferate in hypoxic tumor cores, release phospholipases and cytolysins that directly kill neighboring tumor cells [38]. For instance, clostridial α-toxin lyses cancer cell membranes, while *Salmonella spp.* express bacterial cytotoxins that induce tumor cell apoptosis—effects that mimic those of chemotherapeutic agents and may be harnessed for direct tumor debulking. Moreover, bacterial infection often leads to focal inflammation and coagulative necrosis, further restricting tumor growth and spread. Indeed, intratumoral injection of nontoxigenic clostridial spores has been shown to induce extensive hemorrhagic necrosis and reduce the pool of viable tumor cells [39].

In line with emerging concepts in “tumor microecology-immune regulation”, the engineered bacteria–nanomaterial hybrid systems paradigm leverages the growing understanding that tumors are not sterile entities: Intratumoral microbial communities vary by cancer type and location, influencing both therapeutic responses and resistance, with microbial diversity correlating with patient prognosis. Concurrently, the gut microbiota shapes antitumor immunity at a distance through metabolites and immune modulation, forming a gut–tumor immune axis that substantially affects immunotherapy efficacy [3]. In this context, bacterial programmability and tumor tropism make them ideal living carriers: Genetically engineered strains can deliver and release therapeutic factors directly within tumors, amplifying local-to-systemic immune effects [40]. For instance, introducing the tumor necrosis factor-related apoptosis-inducing ligand (TRAIL) gene into *E. coli* enables intratumoral TRAIL expression, leading to cancer cell apoptosis, illustrating how bacteria can be transformed from passive residents into programmable intratumoral delivery vehicles for cancer therapy [41]. Together, these insights outline a practical approach and specific targets for combining microecological modulation with engineered living delivery systems to advance cancer immunotherapy.

### Selection criteria and representative antitumor bacterial chassis

An ideal chassis for therapeutic bacterial engineering should preferentially accumulate within tumors while sparing healthy tissues. This selectivity leverages bacteria’s natural affinity for the TME: Obligate and facultative anaerobes thrive in hypoxic, nutrient-rich regions with reduced immune surveillance [42]. Spore-forming clostridia, for example, germinate exclusively in necrotic, oxygen-deprived tumor cores, exhibiting highly tumor-selective outgrowth without harming well-oxygenated normal tissues [43]. In contrast, facultative anaerobes such as *Salmonella* possess inherent tumor tropism; they can traverse the bloodstream, infiltrate tumor lesions, and proliferate several orders of magnitude more than in normal tissues, aided by the permissive TME [40]. Collectively, these features confer substantial advantages for precise tumor targeting when selecting bacterial strains for therapeutic applications [44].

Safety attenuation is critical when selecting therapeutic chassis: Strains must maintain sufficient activity while minimizing the risk of severe infections. Highly virulent wild-type strains are unsuitable as carriers; instead, researchers often delete virulence factors or introduce auxotrophies to create attenuated variants with improved biosafety. A well-known example is *VNP20009*, an attenuated *Salmonella enterica* serovar *Typhimurium* derived by disrupting purI (purine auxotrophy) and msbB (lipid A modification), which markedly reduces virulence while preserving tumor-selective behavior. *VNP20009* has advanced to a phase I clinical trial in melanoma patients [45]. An alternative approach involves using naturally safe probiotics as chassis. EcN, a nonpathogenic probiotic lacking many virulence factors found in pathogenic strains, is clinically well-tolerated. Its fully sequenced genome and genetic tractability make it a strong candidate for engineering, positioning EcN and similar low- or nontoxigenic strains as attractive foundations for developing therapeutic engineered bacteria [46].

Strain selection also requires that bacteria be amenable to genetic manipulation and tightly controlled. Preference is given to model organisms with stable genomes and established genetic tools—such as plasmids, promoters, selection markers, and editing methods—that allow for the insertion of heterologous genes, deletion of nonessential loci, and integration of regulatory elements for tunable expression. For example, *E. coli* is among the most extensively studied organisms in molecular biology and is highly amenable to genetic engineering; consequently, many therapeutic constructs utilize *E. coli* as the chassis [46]. Similarly, *S. enterica* serovar *Typhimurium* is easy to culture, supported by well-established attenuated strains, and widely used in drug delivery applications [47]. Additionally, therapeutic constructs often incorporate safety modules, such as temperature-sensitive elements, lac operon-based control, and programmed kill switches, to enable spatiotemporal regulation and biocontainment. These mechanisms ensure that engineered bacteria–nanomaterial hybrid systems remain safe and controllable, activating tumoricidal functions on demand within lesions while being suppressed or eliminated in nontumor tissues.

Under these criteria, representative bacterial chassis differ substantially in oxygen preference, tumor tropism, genetic tractability, immune stimulation, clinical precedent, and safety limitations. To provide a clearer chassis-level comparison, these properties are summarized in Table 1.

**Table 1.** Representative bacterial chassis for engineered bacteria–nanomaterial hybrid systems

Chassis	Colonization features	Engineering/immune features	Translational evidence	Main limitations
*E. coli Nissle* 1917	Facultative anaerobe	Genetically tractable	Probiotic chassis; SYNB1891 tested clinically [46]	Weaker tumor restriction than strict anaerobes
Attenuated *Salmonella*	Facultative anaerobe	Highly engineerable; high payload capacity	VNP20009 and SGN1 tested clinically [40,45]	Residual virulence, inflammation
*Clostridium* spp.	Obligate anaerobe	Spore-based tumor targeting	*C. novyi*-NT evaluated in early clinical studies [43]	Requires hypoxic or necrotic tumors
*Bifidobacterium* spp.	Anaerobe	Probiotic-like safety	APS001F explored in enzyme-prodrug therapy [36]	Slow growth
*Listeria monocytogenes*	Facultative intracellular bacterium	Potent cellular immune activation	CRS-207 and ADXS11-001 tested clinically [159–161]	Intracellular pathogenicity and safety concerns
Other attenuated/probiotic strains	Strain-dependent tropism, motility, and oxygen preference	Variable engineering capacity, payload compatibility, and immune stimulation	Mostly preclinical or early translational	Limited standardization; strain-specific behavior

## Construction Strategies for Engineered Bacteria–Nanomaterial Hybrid Systems in Cancer Treatment

Bacteria provide a living and programmable chassis for tumor targeting, payload delivery, and immune stimulation, but their clinical use is constrained by native virulence, endotoxin-driven inflammation, and systemic dissemination. To balance efficacy with safety, this section discusses construction strategies as an engineering workflow: chassis programming, bacteria–nanomaterial interface construction, payload loading, stimulus response, controlled release, immune output, and safety and manufacturing control.

### External engineering strategies for bacterial modification

External engineering strategies primarily involve surface modifications, including electrostatic interactions, chemical coatings, and biological modifications. Surface modifications impart additional functionalities: electrostatic assembly for efficient nanomaterial loading, chemical coatings for stable control, and biological modifications for enhanced targeting and biocompatibility. These approaches complement genetic engineering, expanding the therapeutic potential of bacteria-based cancer treatments.

#### Electrostatic interactions

**Fig. 4.**
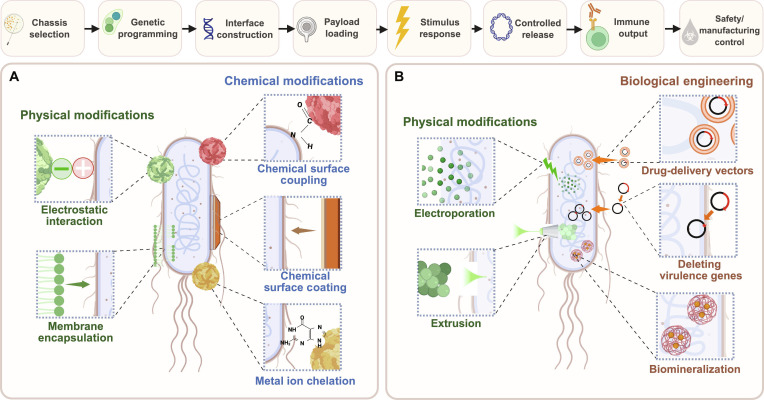
Engineering workflow for constructing bacteria–nanomaterial hybrid systems. The upper workflow summarizes chassis selection, genetic programming, interface construction, payload loading, stimulus response, controlled release, immune output, and safety and manufacturing control. (A) External interface engineering for nanomaterial attachment, payload loading, and stimulus-responsive release. (B) Internal programming strategies for bacterial sensing, therapeutic output, and safety control.

Electrostatic assembly leverages the net negative charge of bacterial envelopes to form stable Coulombic interfaces with cationic nanomaterials, enabling rapid, solution-phase decoration of living cells (Fig. 4A). Under near-physiological conditions (pH 6.0 to 7.5), the outer membranes of Gram-negative species such as *E. coli* and *Salmonella Typhimurium* readily bind positively charged NPs through simple co-incubation, resulting in robust bacterium–NP hybrids. For instance, Zhao et al. [48] used this strategy to adsorb black phosphorus quantum dots (BPQDs) onto *E. coli*, constructing *E. coli*/BPQD hybrids that effectively target hypoxic tumors and mediate photothermal therapy (PTT). Upon exposure to 660-nm laser irradiation, the hybrid generated reactive oxygen species (ROS), disrupted tumor cell membranes, and catalytically promoted catalase release, thereby eliciting antitumor effects.

Beyond direct adsorption, modulation of the bacterial surface potential expands the design possibilities for cationic functionalization. For example, coating the facultative anaerobe *Shewanella oneidensis* MR-1 with biocompatible poly(allylamine hydrochloride) (PAH) reverses its ζ-potential, enabling the subsequent assembly of negatively charged, hyaluronic acid (HA)-coated, and doxorubicin (DOX)-loaded MIL-101 metal–organic framework NPs. This results in the SO@MIL-101-DOX-HA biohybrid, which achieves tumor targeting and controlled release via a cascaded mechanism, leading to a tumor inhibition rate of 78.3% [49]. In another complementary 2-step “electrostatic bridge” strategy, Hu et al. [50] employed a positively charged chitosan interlayer to anchor negatively charged poly(lactic-co-glycolic acid) (PLGA)/indocyanine green (ICG) NPs onto the surface of *E. coli*, thereby constructing a photothermal-responsive platform (E@L-P/ICG). In a colorectal cancer model, this platform accumulated in tumors, where mild laser heating triggered the disassembly of E@L-P/ICG and the coordinated release of the immunostimulatory cytokine LIGHT (TNFSF14) along with tumor-associated antigens (TAAs), ultimately remodeling the tumor immune microenvironment and suppressing tumor progression.

Although charge-mediated assembly is simple and biocompatible, its structural integrity can be compromised in vivo. Factors such as counter-ion screening, high ionic strength, protein corona formation, and hemodynamic shear weaken the electrostatic interface. Excessive cationization may also disrupt membrane-potential homeostasis, impairing bacterial metabolism and motility. These challenges can be mitigated by adjusting surface charge density within a viability-preserving range and by incorporating thin spacer layers to decouple the electrostatic coating from the inner membrane.

#### Chemical surface coating

Polymeric coatings can enhance the chemical robustness, promote immune evasion, and enable programmable release while maintaining bacterial motility and viability. Polymers with abundant functional groups can be tethered to bacterial envelopes through covalent chemistries, such as carbodiimide or click reactions, or via engineered noncovalent interactions. These modifications result in bacterium–nanomaterial hybrids with improved mechanical resilience, reduced nonspecific adhesion, and extended persistence in vivo. Such coatings physically insulate bacteria from the hostile TME, mitigate complement and opsonin deposition, and create diffusion barriers that allow controlled or sustained drug release [51]. Among universal coatings, polydopamine (PDA)—a mussel-inspired catecholamine polymer—forms conformal shells through π–π stacking, hydrogen bonding, and electrostatic adsorption without impairing bacterial functionality [52]. Using oxidative self-polymerization, Chen et al. [53] anchored PDA NPs onto *S. Typhimurium VNP20009*, generating a hybrid with intrinsic tumor tropism and high near-infrared (NIR) photothermal conversion. Notably, the engineered bacteria–nanomaterial hybrid retained its biological functions while enabling precise photothermal ablation of tumor tissues. From a design perspective, coating thickness and crosslinking density must be optimized to balance protection with mass transport. Excessive barrier effects should be avoided, as they can hinder nutrient exchange, disrupt signal transduction, and reduce responsiveness to external stimuli.

#### Biological surface modification

Biological surface modification utilizes molecular recognition and mineralization processes to establish highly specific and multifunctional bacterium–nanomaterial interfaces. These strategies offer opportunities to combine the inherent targeting and metabolic properties of bacteria with exogenous functional modules, thereby broadening therapeutic versatility.

Biorecognition approaches rely on selective, high-affinity interactions to guide the assembly of hybrid constructs. A variety of targeting ligands—including antibodies, peptides, aptamers, and small-molecule receptor ligands—can be conjugated to bacterial surfaces to enhance tumor specificity. These ligands are typically selected based on TAAs or overexpressed cell-surface markers. Several reviews have summarized the landscape of tumor-targeting ligands and their integration into microbial or nanocarrier systems, highlighting their potential to refine bacterial precision in cancer therapy [54] (refer to the previous section).

In parallel, in situ biomineralization leverages biologically mediated inorganic deposition to generate protective or functional coatings on bacterial envelopes. Species such as *Bacillus subtilis* and *S. oneidensis* can sequester metal ions and enzymatically reduce them to their elemental forms via nitrate/nitrite reductases, sulfite/sulfate reductases, NADH [reduced form of nicotinamide adenine dinucleotide (oxidized form)]/NADPH (reduced form of nicotinamide adenine dinucleotide phosphate)-dependent oxidoreductases, and metal-binding peptides such as metallothioneins and phytochelatins. These processes initiate the nucleation of metallic NPs such as Au, Ag, or Cu with minimal impact on bacterial viability. For example, *E. coli* MG1655 expresses NADPH-dependent surface reductases that can reduce Au^3+^ to Au^0^, resulting in the in situ formation of gold NPs directly on the bacterial envelope [55–57]. This case illustrates how intrinsic enzymatic pathways can be exploited to template nanomaterial deposition with spatial precision.

Exploiting extracellular electron transfer in metal-reducing bacteria further enhances the biomineralization toolbox. Electrons derived from bacterial metabolism can be transferred across membranes to terminal metal ions, promoting nucleation and hybrid material formation. For instance, *S. oneidensis* MR-1, a chassis with strong electron-shuttling capacity, has been combined with FeS NPs to create a self-mineralizing platform (SO@FeS). In this system, the bacterial metabolism of intratumoral lactate provides a continuous electron supply to sustain the Fe^3+^/Fe^2+^ redox cycle, while FeS NPs release Fe^2+^ and H₂S under acidic tumor conditions, initiating chemodynamic therapy (CDT) and gas therapy. The coordinated interaction between bacterial metabolism and nanomaterial reactivity synergistically remodels the TME and inhibits tumor growth [58]. Similarly, *Shewanella algae K3259* has been used as a metal-reducing chassis for Au biomineralization, where photoexcited electrons from Au NPs are funneled through its electron-transfer pathways, driving in situ tetrodotoxin biosynthesis and enhancing antitumor activity.

Collectively, biological surface modification strategies—ranging from ligand-directed recognition to biomineralization—enable the development of highly specific, responsive, and multifunctional bacterial platforms. By integrating targeting ligands with bacterial tropism and combining metabolic electron transfer with mineralized nanomaterials, these strategies expand the design space for engineered bacteria–nanomaterial hybrid systems, opening new avenues for precision cancer therapy.

### Internal engineering strategies for bacterial modification

In contrast to external modifications, internal engineering strategies reprogram bacteria from within to enhance their stability, biosafety, and functional adaptability within the TME. Key approaches include synthetic biology, which allows for precise genetic editing and modular circuit design to regulate bacterial behavior; electroporation, which transiently permeabilizes the cell membrane to facilitate the introduction of exogenous genes or functional cargo; and biomineralization, which harnesses bacterial metabolic pathways to nucleate inorganic nanostructures with defined optical, magnetic, or acoustic properties. Together, these strategies provide a solid foundation for improving the precision, controllability, and therapeutic efficacy of engineered bacteria–nanomaterial hybrid systems in cancer immunotherapy [59].

#### Synthetic biology

Synthetic biology offers a powerful framework for rationally reprogramming bacterial functions. Advances in genome editing and modular circuit design now enable the integration of exogenous genes and the precise modification of endogenous loci, allowing for the reshaping of bacterial traits as needed [60]. Using these tools, synthetic gene circuits are constructed to provide bacteria with programmable sensing, computation, and actuation capabilities. These circuits can precisely regulate bacterial growth, therapeutic payload expression, and release with spatiotemporal accuracy.

Plasmids remain the most widely used scaffolds for constructing such systems. As circular DNA vectors, they accommodate customizable modules, including promoters, open reading frames, replication origins, and selectable markers. Stable transformation allows for the controlled expression of proteins, peptides, antibodies, antibiotics, and enzymes within the TME, thereby enhancing bacterial functionality and therapeutic efficacy [61,62]. In parallel with vector engineering, regulatory design is crucial. Strategies include promoter gating responsive to hypoxia, pH, or quorum signals; circuit layering to implement logic-based operations; and biocontainment modules, such as kill switches or auxotrophies, to balance efficacy and safety. These features collectively enhance the precision and controllability of engineered bacteria–nanomaterial hybrid systems.

An attenuated *VNP20009* strain was engineered to incorporate a synchronized lysis circuit (*SLC*) alongside constitutive expression of the cytokine LIGHT. This quorum-sensing-regulated system enables the conditional, intratumoral release of LIGHT upon reaching a critical bacterial density, thus enhancing antigen presentation, promoting the maturation of tertiary lymphoid structures, and activating CD8^+^ T cell-mediated antitumor immunity while maintaining normal bacterial growth kinetics and an improved safety profile [61]. In multiple tumor models, *SLC-VNP20009* effectively suppressed tumor growth and remodeled the gut microbiota, demonstrating the therapeutic potential of combining synthetic circuits with attenuated bacterial chassis. Additionally, Wang et al. [63] engineered a clustered regularly interspaced short palindromic repeats and deactivated CRISPR-associated protein 9 (CRISPR-dCas9) platform, termed OMV-C9I12, to coexpress the chemokine CXCL9 and interleukin-12 (IL-12) in the TME. This dual-gene regulation strategy enhanced intratumoral T cell infiltration, amplified antitumor immunity, and achieved marked tumor suppression in preclinical models. This study highlights the potential of CRISPR-dCas9 technologies to precisely reprogram bacterial outputs and deliver combinatorial immunomodulatory cues, thereby broadening the therapeutic possibilities of synthetic biology-driven bacterial platforms.

Overall, synthetic biology-driven construction provides a systematic approach to high-efficiency payload loading, precise intratumoral delivery, and enhanced biosafety while preserving the intrinsic tumor tropism of living bacterial carriers.

#### Electroporation

The impermeability of bacterial membranes and physiological barriers often limits the intracellular loading of therapeutic payloads. Reversible electroporation offers a solution by delivering short electrical pulses that transiently permeabilize the bacterial envelope. This process allows otherwise impermeant small molecules, drugs, or liposomes to enter the cytoplasm, after which the membrane reseals (Fig. 4B).

In a representative study, Xie et al. [64] applied electroporation to EcN, successfully introducing 5-fluorouracil (5-FU) and zoledronic acid. As noted in a recent review by Din et al. [65], electroporation can facilitate the efficient delivery of plasmids or DNA fragments into bacterial chassis, enabling stable gene integration or transient expression of therapeutic payloads. While electroporation may slightly compromise membrane integrity and cellular fitness, it remains a practical strategy for intracellular encapsulation when controlled within a viability-preserving window.

#### Biomineralization technology

Harnessing native transport systems and biomineralization capacity offers a unique method for intracellular payload loading. This strategy maintains the living carrier concept while enhancing lesion specificity. For instance, glucose polymer (GP)-functionalized, ICG-loaded silicon NPs (GP-ICG-SiNPs) can be selectively internalized by facultative anaerobes such as attenuated *S. Typhimurium VNP20009* or *E. coli ATCC 25922* [66]. This approach facilitates efficient traversal of the blood–brain barrier and precise targeting of glioblastoma.

Additionally, intracellular biomineralization can be programmed to synthesize inorganic NPs inside bacterial carriers. This process enhances antitumor efficacy while minimizing systemic exposure. A notable example involves using EcN as a living bioreactor to produce tellurium nanorods (TeNRs), resulting in a biohybrid termed Te@EcN [67]. Although TeNR biosynthesis reduced bacterial proliferation, EcN maintained stable and prolonged intratumoral accumulation. Notably, the attenuated probiotic chassis offered superior biosafety compared to highly proliferative live-bacteria therapies.

Internal engineering strategies provide powerful tools to reprogram bacterial carriers from within. Synthetic biology enables modular control over sensing, payload expression, and safety; electroporation offers a direct method for intracellular loading of therapeutic molecules; and biomineralization technologies allow bacteria to function as living bioreactors for producing functional nanomaterials. Collectively, these approaches enhance precision, efficacy, and biosafety, expanding the therapeutic potential of engineered bacteria–nanomaterial hybrid systems in cancer treatment.

### Combined internal and external engineering strategies for bacterial platforms

Single-modality engineering often falls short of delivering high payloads, precise control, and robust efficacy within the complex TME. To address these limitations, integrated internal–external strategies are employed to create hierarchical biohybrid platforms with considerable translational potential [68]. Internal programming provides chassis attenuation, tumor tropism, environment-gated expression, and programmed release. External interfaces introduce stimulus responsiveness, imaging or diagnostic capabilities, and orthogonal functional modules. This dual-layer design links navigation, activation, and release, enhancing stability, barrier traversal, and targeting, all while maintaining control.

Interface stability must be evaluated under physiological rather than only formulation conditions. Electrostatic assemblies may weaken after ionic screening, serum protein corona formation, and blood flow shear, whereas covalent or bioorthogonal linkers generally improve payload retention but may reduce membrane flexibility if overused. Coatings such as polyethylene glycol (PEG), cell-membrane mimics, or thin hydrogels can limit opsonization and immune clearance, but their thickness should be controlled to preserve bacterial motility, nutrient exchange, and signal responsiveness. Bacterial proliferation can also dilute surface-bound cargos over time, making release kinetics and payload retention key parameters for in vivo design [68].

**Fig. 5.**
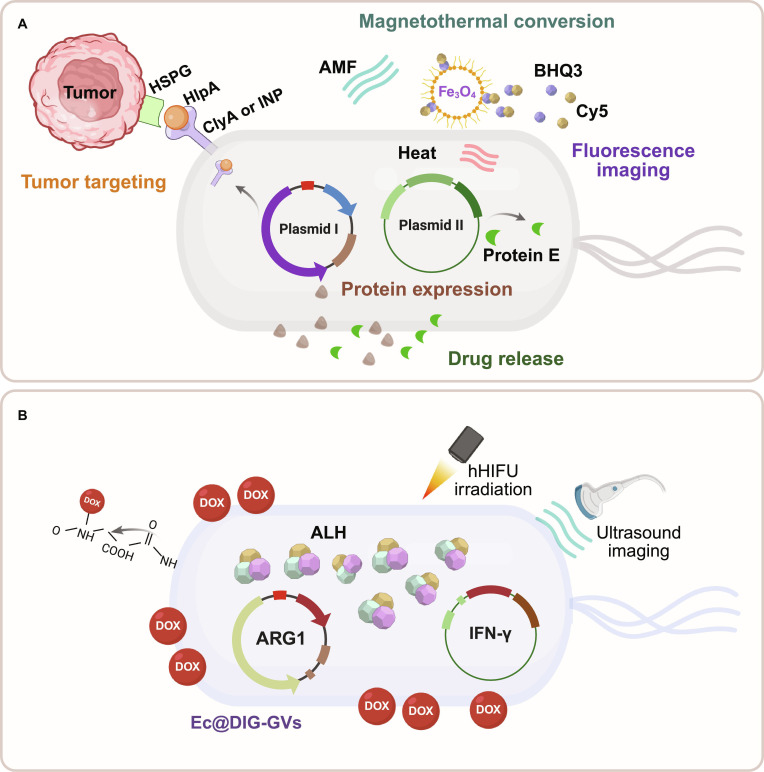
Remotely controlled engineered bacteria–nanomaterial hybrid systems for tumor-targeted imaging and therapy. (A) Genetically engineered *E. coli* coated with Fe_3_O_4_@lipid nanocomposites act as a tumor-homing bacteria, converting alternating magnetic field signals into heat to activate lysis protein expression via a heat-sensitive promoter. (B) Schematic of Ec@DIG-GVs, genetically engineered *E. coli* MG1655 carrying arginase 1 (ARG1) and a hyperthermia-responsive interferon-γ (IFN-γ) circuit, surface-linked with DOX, enabling ultrasound imaging and high-intensity focused ultrasound (HIFU)-triggered gene expression and drug release for synergistic antitumor immunity. AMF, alternating magnetic field; HSP, heat-shock promoter; HlpA, histone-like protein A; INP, ice nucleation protein; ClyA, cytolysin A; BHQ3, Black Hole Quencher 3; Cy5, cyanine 5; ALH, acid-labile hydrazone linker.

Representative studies illustrate how internal circuits and external material interfaces can be integrated for coordinated control. In a hyperthermia-responsive engineered *E. coli* platform, genetically engineered *E. coli* carrying a hyperthermia-responsive circuit was surface-linked with DOX and gas vesicle (GV) modules, enabling ultrasound imaging and heat-triggered therapeutic output (Fig. 5B) [69]. In a magnetically guided programmed bacterial platform, magnetic guidance can be integrated with a programmed chassis to assist with barrier crossing and spatial control (Fig. 5A). These examples demonstrate the value of combining internal programming with externally controllable interfaces while also underscoring the need to evaluate interface stability, bacterial fitness, and payload retention in vivo.

Overall, internal and external engineering strategies provide versatile approaches to enhance stability, targeting, and functional precision of bacterial platforms. These approaches lay the foundation for constructing programmable and clinically relevant biohybrids. Building on this foundation, the next critical step involves rational design and systematic optimization to refine bacterial performance, balance efficacy with safety, and accelerate clinical translation.

## Rational Design of Engineered Bacteria–Nanomaterial Hybrid Systems

### Surface engineering of engineered bacterium–physically responsive nanoplatforms

**Fig. 6.**
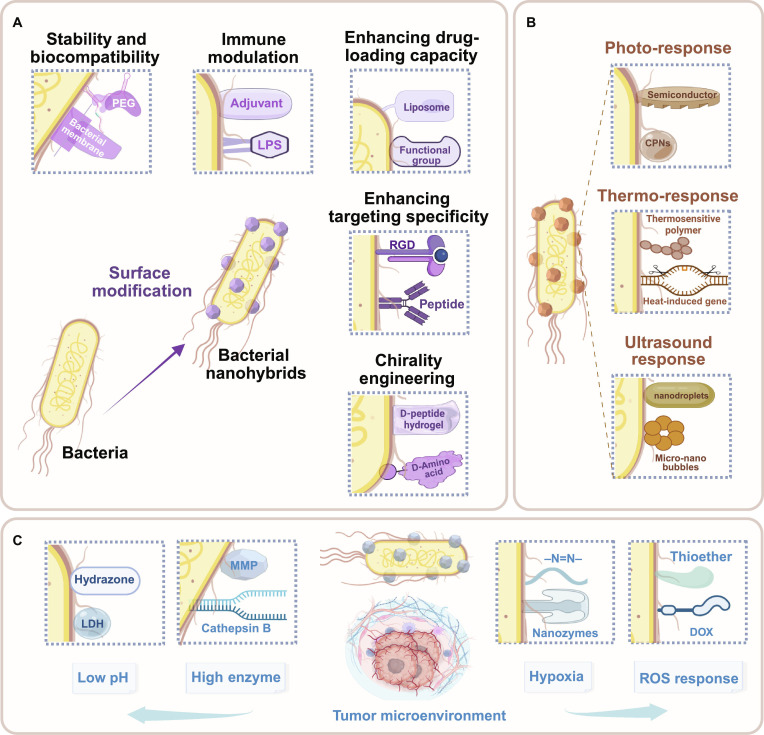
Surface design and optimization of engineered bacteria–nanomaterial hybrid systems. (A) Surface engineering strategies. (B) Physical responsiveness via surface modification. (C) Tumor microenvironment responsiveness via surface modification. LPS, lipopolysaccharide; LDH, lactate dehydrogenase.

Within intelligent, physically responsive nanoplatforms that synergize with engineered bacteria for anticancer therapy, surface modification and functionalization go beyond traditional objectives such as ensuring colloidal stability, low immunogenicity, and accurate targeting (Fig. 6A). These modifications create a critical interface that links external physical-stimulus signal transduction with bacteria-to-nanomaterial integration. By precisely assembling photo-, magneto-, or sono-responsive moieties alongside degradable “gating” linkers on the NP surface, on-demand drug or gene release can be triggered upon the application of corresponding external fields. Simultaneously, grafting antibodies, nucleic acid aptamers, peptides, or small-molecule ligands directs the system toward tumor-specific antigens (Table 2). Alternatively, biomimetic cloaking with autologous bacterial membranes or the outer membranes of targeted bacterial strains leverages the microorganisms’ innate chemotaxis and homotypic adhesion, facilitating deep tumor penetration and “self-recognition” targeting. Together, these strategies create a 3-step synergy—bacteria-driven navigation, physical-stimulus activation, and precision payload delivery—within the TME, laying a basis for the development of high-efficiency stimulus-responsive modules, optimization of the bacterium–nanomaterial interface, and thorough safety evaluation for clinical translation.

**Table 2.** Surface functionalization and key design parameters of physically responsive nanomaterials. Key design parameters for these modules include particle size, surface charge, coating thickness, loading efficiency, photothermal or ROS-generating capacity, acoustic or magnetic responsiveness, biodegradability, and in vivo interface stability.

Category	Feature	Surface modification materials	References
Thermosensitive materials	Melting at >41 °C	DPPC	[86] [170]
		DSPE-PEG2K	[170]
Immunoadjuvants	Activate dendritic cells (DCs) and present antigens to T cells	CpG	[171]
	Block PD-1/PD-L1 pathway to activate T cells		
Targeting ligand-antibody	Antibody modification—recognition of tumor markers	LyP-1 targets and binds to p32	[172]
		d-Pen or l-Pen binds specifically to ferritin	[173]
		d/l-Aspartic acid and heat shock protein (HSP90) targeted binding	[174]
	Peptide—recognizes tumor markers	RGD peptide (peptide containing the Arg-Gly-Asp sequence)—recognizes integrins	[78] [175]
	Nucleic acid—identifying tumor markers	Aptamers—obtained through in vitro screening (SELEX technology)	[176]
	Small molecule ligands—recognize specific ligands	Folic acid (FA)—recognizes folic acid receptors	[79]
	Biological adhesion molecules	Hyaluronic acid (HA)—binds to the CD44 receptor	[177]
pH targeting	Low pH responsiveness	PEG-b-PAA	[178]
Enzyme targeting	Recognition of MMPs	PLGMWSR	[179]
		PLGLWA	
	Recognition of Cathepsin B	GFLG	
		GGFG	
	Recognition of Caspases	DEVD	
		VAD	
Hypoxia targeting	Hypoxic environment	Ferritin	[180]
		Transferrin	[181]
Passive targeting	EPR	PEG	[182]
		PLGA	[183]
		PGA	[183]
		PEG-PC	[184]
Polymer–biomolecular composite materials	Polymer–antibody composite	PEGylated antibody—targeting HER2/EGFR	[185]
	Polymer–peptide composite materials	PLGA combined with RGD peptide—targeting integrin αvβ3	[185]
	Polymer–oligonucleotide complex	PEI-encapsulated oligonucleotides or aptamer modified	[186]
	Polymer–biological ligand composite	PLGA–folic acid-targeted folate receptor	[187]
	Polymer–enzyme complex	pH-sensitive polymer-hyaluronidase—targets HA	[188]
	Other polymer blends	Polylactic acid–glycolic acid	[189]
Surfactant	Cationic surfactant	Cetyltrimethylammonium bromide (CTAB) targeting negatively charged tumor cells	[190]
	Amphiphilic surfactant	PEG–PLA copolymer	[191]
Regulate autophagy/promote apoptosis/enhance photothermal performance/improve the biocompatibility of nanomaterials/increase the uptake by tumor cells	Chiral molecule	d/l-Cysteine	[192]
		l-Tryptophan	[193]
		l-Methionine	[194]

DCs, dendritic cells; CpG, cytosine–phosphate–guanine; DPPC, 1,2-dipalmitoyl-sn-glycero-3-phosphocholine; DSPE-PEG2K, 1,2-distearoyl-sn-glycero-3-phosphoethanolamine–polyethylene glycol 2000; HSP90, heat shock protein 90; SELEX, systematic evolution of ligands by exponential enrichment; FA, folic acid; CD44, cluster of differentiation 44; PEG-b-PAA, polyethylene glycol-block-poly(acrylic acid); PGA, poly(glycolic acid); PEG-PC, polyethylene glycol–phosphatidylcholine; HER2, human epidermal growth factor receptor 2; EGFR, epidermal growth factor receptor; PEI, polyethyleneimine; CTAB, cetyltrimethylammonium bromide; PEG–PLA, polyethylene glycol–poly(lactic acid)

#### Enhancing stability and biocompatibility

Surface PEGylation remains the standard approach for extending the blood-circulation half-life of nanomaterials and reducing nonspecific protein adsorption. Single-molecular-layer super-resolution imaging has revealed that excessively long PEG chains can entomb targeting ligands within the hydration shell, compromising receptor accessibility. Achieving an optimal balance between chain length and ligand exposure is, therefore, essential [70]. Incorporating PEG derivatives onto the membrane of the photosynthetic bacterium *Rhodopseudomonas palustris* markedly reduces macrophage phagocytosis and improves hemodynamic stability. Concurrent conjugation of an anti-programmed death ligand 1 (PD-L1) antibody provides immune checkpoint blockade. Following NIR laser irradiation, these PEGylated biohybrids induce photothermal-immune tumor ablation in a murine colon cancer model, highlighting the clinical potential of the “surface PEGylation + photophysical stimulation” strategy [71].

Biomimetic coating strategies can enhance the in vivo performance of these platforms. By cloaking bacterial surfaces with natural cell membranes—such as those derived from red blood cells, platelets, or even cancer cells—researchers can bestow these living therapeutics with immune evasion capabilities, prolonged systemic circulation, and improved biocompatibility [72]. These cell membrane cloaks act as biological camouflage, reducing complement activation and phagocytic clearance while preserving bacterial functionality. This enables more effective tumor colonization and therapeutic action [73].

Polymeric coatings, particularly PDA and biodegradable PLGA, have been applied to improve platform stability and therapeutic output. By forming conformal protective shells, these polymers improve mechanical durability, minimize nonspecific surface interactions, and protect bacteria from premature immune elimination. Such polymer–bacteria hybrids represent a flexible and effective strategy to optimize bacterial function in vivo, thereby expanding their translational potential in cancer therapy.

Hydrogel and alginate encapsulation provide another approach to improve local retention and sustained therapeutic output [74]. By embedding living cells within biocompatible, semi-permeable matrices, these systems protect bacteria from rapid immune clearance while maintaining nutrient exchange and metabolic activity. This encapsulation also allows for localized retention at tumor sites and facilitates sustained or stimuli-responsive release of therapeutic agents [75].

Overall, PEGylation, biomimetic cloaking, polymeric coatings, and hydrogel or alginate encapsulation offer complementary strategies to modulate circulation time, immune shielding, mechanical stability, and local retention. These designs must balance protection with bacterial viability, motility, nutrient exchange, and stimulus responsiveness.

#### Enhancing targeting specificity

Anchoring antibodies, peptides, aptamers, or small molecules onto NP surfaces—via covalent or noncovalent chemistry—remains a cornerstone strategy for enhancing active targeting. Weak interactions such as electrostatic attraction, hydrophobic forces, hydrogen bonding, and π–π stacking allow for rapid fabrication and controllable ligand release, whereas robust covalent linkages provide stability against the complex in vivo environment [76]. For instance, tLyP-1 peptide-modified ICG@HSA-Azo-HP penetrates triple-negative breast cancer tissue deeply through the CendR pathway and generates potent photothermal heating upon laser irradiation [77]. Similarly, the incorporation of arginine–glycine–aspartic acid (RGD) peptides [78], folic acid [79], and other targeting ligands markedly increases tumor tropism of nanomaterials. Aptamer-based nanoplatforms targeting tumors overexpressing receptors such as MUC1, HER2, EGFR, EpCAM, or PSMA also exhibit lower immunogenicity and easier conjugation than their antibody-based counterparts [80].

Coating NP cores with cancer cell, platelet, or stem cell membranes confers homotypic adhesion, immune evasion, and prolonged systemic circulation. For example, Renca cell membrane-camouflaged CuO@Gd₂O₃ yolk-shell NPs exploit homotypic recognition to penetrate clear cell renal cell carcinoma (ccRCC) and elicit ICD through the combined actions of cuproptosis and CDT [81]. Similarly, platelet membrane-coated copper-doped polypyrrole-BPTES particles trigger simultaneous cuproptosis and ICD in glutathione (GSH)-rich environments [82]. Systematic characterization indicates that membrane integrity and fluidity critically determine tumor accumulation efficiency, with a 3-step “adsorption–rupture–fusion” assembly mechanism. Incorporation of solubilizing phospholipids increases the fraction of fully coated NPs from 6% to approximately 23% [83].

In recent years, engineered bacteria–nanomaterial hybrid systems and their membrane-derived vesicles have provided a novel route for deep tumor targeting [84]. The model strain *Magnetospirillum gryphiswaldense*, when loaded with drug-laden liposomes, is capable of penetrating hypoxic zones in melanoma, markedly inhibiting tumor growth, and eliciting strong immune activation [85]. Magnetically guided or chemotaxis-regulated bacterium–NP hybrids can evade reticuloendothelial clearance. Once within the TME, these hybrids release sonosensitizers, photosensitizers, or immunostimulatory cargos, triggering a cascading “self-propelled infiltration–physical activation–immune amplification” response [86]. Additionally, bacterial extracellular vesicles, modified with 1,2-distearoyl-sn-glycero-3-phosphoethanolamine (DSPE)–PEG–RGD, can deliver iron–DOX complexes to induce ferroptosis and activate the STING pathway, demonstrating favorable long-circulation profiles and tumor tropism [87].

In summary, precise ligand engineering ensures high-affinity engagement with tumor cell receptors, biomimetic membrane cloaking provides “self” camouflage and homotypic adhesion, and engineered bacterium propulsion harnesses intrinsic chemotaxis and microenvironment-responsive activation to achieve deep tissue penetration and spatiotemporally controlled payload release. These 3 modalities, when combined, create a hierarchical targeting architecture that has led to pronounced drug accumulation, immune activation, and tumor suppression across multiple murine models.

#### Immune modulation

Immune activation by these hybrids usually follows an ordered sequence. After the TME provides a permissive niche for targeted delivery, seeding, and growth of the bacterial chassis, the nanomaterial module is activated by light, ultrasound, magnetic fields, or tumor-derived cues. Local heating, ROS generation, cavitation, or redox reactions then injure tumor cells and promote ICD. Together, bacterial PAMPs and tumor-derived damage-associated molecular patterns (DAMPs) and tumor antigens drive antigen uptake and presentation, leading to T cell priming, effector cell infiltration, and, in some models, immune memory or improved responses to checkpoint blockade.

The 2 modules contribute to this immune cascade in different ways. Bacteria mainly function as living adjuvants: Their surface structures and nucleic acids provide PAMPs that engage pattern-recognition pathways and initiate local innate immune activation. By contrast, the material module mainly supplies inducible tumor injury. Photothermal, photodynamic, sonodynamic, magnetothermal, and chemodynamic nanomaterials generate heat, ROS, cavitation, or redox stress, thereby promoting ICD, DAMP release, and tumor-antigen exposure. In this sense, bacteria provide the adjuvant signal, whereas nanomaterial-induced damage provides the antigenic and danger signals required for adaptive immunity.

Importantly, local inflammation or immune-cell infiltration should not be equated with durable antitumor immunity. Where possible, immune remodeling should be evaluated by stronger adaptive endpoints, including antigen presentation, cytotoxic T cell activation, memory T cell formation, tumor rechallenge protection, or improved response to PD-1/PD-L1 or cytotoxic T lymphocyte-associated protein 4 (CTLA-4) blockade. The synchronous delivery of tumor antigens and adjuvants via nanocarriers markedly enriches antigens within antigen-presenting cells (APCs), enhances the CD8^+^ T cell response, and reduces systemic toxicity. Peptide-, DNA-, mRNA-, and cell-derived nanovaccines have demonstrated the “colocalized-delivery” advantage in multiple tumor models, exhibiting superior immunogenicity and safety profiles in animal studies and early-phase clinical trials [88]. To avoid the risks of unchecked immune stimulation, smart nano-immunomodulators incorporate pH-, ROS-, laser-, or ultrasound-cleavable “gating” structures that restrict adjuvant or cytokine release to the TME or to an externally applied trigger, thus preserving overall immune homeostasis [89].

Anchoring the photothermal dye cypate to the engineered probiotic EcN-cypate enables enhanced penetration into hypoxic tumor niches, further augmented by hyperbaric oxygen. Upon NIR-II laser irradiation, this construct induces ICD, recruiting additional immune effector cells and delivering a combined photothermal-immune “double-strike”, providing durable memory protection in a murine melanoma model [90]. Bacterial OMVs, which are naturally enriched with PAMPs such as LPS and peptidoglycan, act as built-in adjuvants that activate TLR/nucleotide-binding oligomerization domain-like receptor (NLR) signaling. After genetic and chemical engineering, OMVs can efficiently load small-molecule drugs, metal ions, or small interfering RNA (siRNA), thereby initiating an “innate priming-adaptive amplification” immune cascade. OMV-derived nanoplatforms have shown strong synergistic potential in multimodal therapies, including phototherapy, chemotherapy, and ferroptosis/cuproptosis strategies [91].

#### Enhancing drug loading capacity

Recent advances in programmable synthesis enable precise modulation of NP composition, surface charge, and stimuli-responsive groups, markedly enhancing drug-to-carrier ratios and providing fine control over release kinetics. Gold nanoclusters (AuNCs) leverage surface plasmon resonance to achieve high-density drug adsorption while imparting intrinsic photothermal functionality. Magnetic nanocarriers, through optimization of magnetic moment and surface functional groups, achieve longer in vivo half-lives and higher payload efficiencies, collectively enhancing chemotherapeutic and gene-delivery outcomes [92]. Liposomes and polymeric micelles, with their “hydrophobic core + hydrophilic corona” architecture, consistently achieve >10 wt % loading for both small molecules and nucleic acids. Their passive or active targeting strategies markedly reduce cardiotoxicity [93]. Live bacteria and their derivatives provide a high-capacity chassis for transporting poorly soluble or labile agents. Magnetosome chains, naturally embedded within a lipid–protein bilayer, carry macromolecules such as antibodies and siRNA with high payloads and excellent biocompatibility [94]. Genetically and chemically engineered OMVs, rich in PAMPs, can densely load small molecules or nucleic acids, retaining circulation stability and enabling the synchronous delivery of “adjuvant + drug” within the TME [95].

#### Chirality engineering as an emerging interface strategy

Chirality—the property of an object that cannot be superimposed on its mirror image—can be imparted to NPs through the grafting of chiral molecules onto their surfaces or by constructing intrinsically chiral nanostructures [96]. These chiral motifs endow the particles with distinctive physicochemical and biological characteristics, enhancing biocompatibility and enabling highly specific recognition of biomacromolecules and tumor cells. In the context of bacteria–nanomaterial hybrids, these effects are relevant because chirality may influence bacterial attachment, tumor cell recognition, cellular uptake, photothermal conversion, and immune activation [96]. Chiral surface ligands can improve aqueous stability, modulate interactions with tumor cell membrane proteins, and alter cellular uptake or stress responses [97]. Under circularly polarized light (CPL), selected chiral nanostructures also show enantioselective optical absorption and enhanced photothermal or catalytic responses, supporting their potential as physical-response modules [98].

More directly related to the present review, several studies have begun to combine chiral nanomaterials with bacterial carriers or bacterial derivatives. Peptide-mediated seed growth inside engineered *E. coli* produces chiral gold NPs in situ while retaining the bacterium’s intrinsic chemotaxis and deep-penetration advantages, enabling an integrated “self-propelled delivery–CPL-triggered” therapeutic platform. Electrostatic self-assembly of l- and d-chiral NPs on the surfaces of *Salmonella* or *Bifidobacterium* markedly improves tumor accumulation and penetration depth, with localized photothermal or ROS generation further amplifying tumor necrosis. OMVs naturally display a d-peptide and LPS chiral microenvironment. Deletion of virulence genes and genetic insertion of tumor antigens or chiral ligands allow OMVs to function as both adjuvants and drug carriers, eliciting robust memory T cell responses and markedly improving safety profiles in murine models.

Although chirality has not yet been systematically explored in fully integrated live bacteria–nanomaterial hybrids, these studies suggest that it may serve as a useful interface feature for tuning bacterial coupling, tumor cell interaction, immune activation, and stimulus responsiveness.

Because different nanomaterial classes are difficult to compare solely by composition, we further summarize the core design parameters that should be reported for physically responsive modules in bacteria–nanomaterial hybrid systems (Table 3).

**Table 3.** Core design parameters for physically responsive nanomaterial modules

Parameter category	Key readouts	Design relevance
Physicochemical properties	Size, PDI, ζ-potential	Loading, dispersion, tumor penetration, reproducibility
Interface design	Coating material, thickness, coupling chemistry	Motility, nutrient exchange, payload retention
Cargo behavior	Loading efficiency, retention, leakage	Payload availability and off-target toxicity
Activation performance	PCE, ROS yield, acoustic or magnetic response	Imaging, remote activation, ICD, immune stimulation
In vivo stability and clearance	Serum/shear stability, bacterial growth, degradation, clearance	Biohybrid fitness, release kinetics, systemic safety

These parameters are not independent; for example, increasing coating thickness may improve payload retention but reduce bacterial motility, nutrient exchange, and stimulus responsiveness. Therefore, future studies should report these parameters together with bacterial viability, motility, and in vivo retention to improve cross-study comparability.

### Externally triggered integration of engineered bacteria–nanomaterial hybrid systems

In intelligent, physically responsive platforms synergized with engineered bacteria, the primary goal is to anchor photo-, magneto-, and sono-sensitive modules securely on the bacterial surface (Fig. 6B). Photothermal dyes and magnetothermal NPs can be covalently grafted via 1-ethyl-3-(3-dimethylaminopropyl) carbodiimide/N-hydroxysuccinimide (EDC/NHS) chemistry or “click” reactions; metal–organic frameworks or polymeric or lipid shells can be electrostatically layered; bioorthogonal coupling via SpyTag-SpyCatcher or biotin–avidin bridges is also utilized; and direct fusion of liposomes or OMVs with the bacterial membrane allows for ultrahigh loading densities. These physical modules are then paired with TME-responsive bonds—such as acidic pH, excess ROS, and hypoxia—enabling a multistage amplification strategy of “navigation first, payload release second, immune potentiation third”. The combination of photothermal, photodynamic, magnetothermal, and sonodynamic effects enhances tissue penetration, intensifies cytotoxicity, and facilitates real-time imaging-guided therapeutic feedback [99]. Modality selection should therefore match tumor depth, device accessibility, bacterial viability, and safety requirements. Light-triggered PTT/photodynamic therapy (PDT) offers high spatial precision and mature optical instrumentation but is most suitable for superficial, endoscopic, or intraoperative lesions because of limited penetration and, for PDT, oxygen dependence. Ultrasound/sonodynamic and magnetothermal strategies are more compatible with deep tumors, although excessive cavitation, heating, or magnetic loading may impair bacterial fitness. CDT avoids external devices and exploits acidic or redox-active tumor niches, but its efficacy depends on local tumor chemistry and may cause nonspecific oxidative stress.

Overall, modality selection should be matched to tumor depth, accessibility to external devices, bacterial viability, and safety requirements. Light-triggered PTT/PDT provides high spatial precision but is mainly suitable for superficial or endoscopically accessible lesions. Ultrasound- and magnetic field-based strategies are more compatible with deep tumors, whereas CDT exploits endogenous tumor chemistry but depends strongly on local redox and acidic conditions.

#### Thermo-responsive nanomaterial integration

Thermo-responsive systems leverage external heating—such as NIR irradiation or alternating magnetic fields—or exploit the hyperthermic TME to reach 40 to 45 °C, triggering phase transitions, pore dilation, or cleavage of carrier linkages to release drugs on demand and locally sensitize the tumor. Classical thermosensitive polymers, such as poly(N-isopropylacrylamide) (PNIPAM) and poly(N-isopropylacrylamide-co-acrylic acid) (PNIPAM-co-AA), transition from hydrophilic to hydrophobic near their lower critical solution temperature, producing a “shrink-and-squeeze” effect to expel cargo [100]. Similarly, an upper critical solution temperature (UCST)-type poly(ethylene glycol)-block-poly(acrylamide-co-acrylonitrile-co-vinylimidazole) (mPEG-PAAV) copolymer co-loaded with IR780 and DOX mediates chemotherapy and photothermal ablation of primary breast tumors and pulmonary metastases under NIR-II laser irradiation [101,102]. The integration of thermo-responsive modules with engineered bacteria–nanomaterial hybrid systems enhances spatial selectivity and amplifies immune effects. For example, gold-nanorod-decorated EcN engineered with a heat-inducible gene switch expresses IL-12 when NIR-induced heating raises the local temperature to 42 °C; combined with regional PTT, this platform achieves >90% tumor inhibition in murine models [103]. In a dual-mode acoustic-thermal construct, GV-bearing *E. coli* undergoes focal-ultrasound-induced warming that releases embedded Cu^2+^/ICG, generating synergistic sonodynamic, photothermal, and cuproptotic effects, while GVs provide real-time ultrasound imaging guidance [69].

#### Photo-responsive nanomaterial integration

**Fig. 7.**
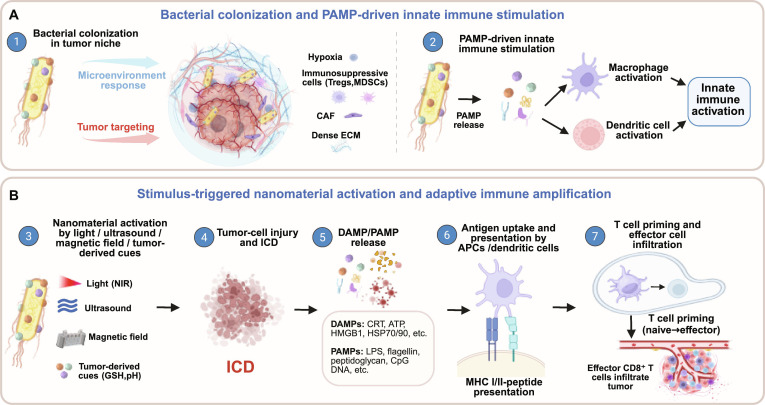
Stepwise antitumor immune activation by engineered bacteria–nanomaterial hybrid systems. (A) Bacterial colonization and PAMP-driven innate immune stimulation within the tumor microenvironment. (B) Stimulus-triggered nanomaterial activation induces tumor cell injury, ICD, DAMP release, antigen presentation, T cell priming, and effector cell infiltration, with downstream potential for immune memory and synergy with immune checkpoint blockade.

The acidic TME of solid malignancies functions as an intrinsic “switch” (Fig. 7A). A saffron-dye/ferrous-ion probe dissociates under pH < 6.8 and, upon NIR-II irradiation, instantaneously releases Fe^2+^, initiating Fenton-like CDT and inducing site-specific oxidative stress [104]. Liposomes co-encapsulating IR808 and loxoribine undergo photothermally driven shell rupture, liberating the TLR7 agonist; this synergy between PTT and immune adjuvants markedly suppresses both primary and metastatic lesions [105]. Live bacteria provide active chemotaxis and deep tissue penetration, imparting self-propulsion to photo-responsive systems. EcN@INX-2, formed by self-assembling an aggregation-induced emission luminogen (AIEgen) onto the surface of EcN, delivers concurrent PTT and PDT under NIR-II illumination, while the bacterial chassis potentiates immune activation, achieving >90% tumor inhibition [106]. In another study, upconversion NPs convert NIR light to blue light, driving a heat-sensitive promoter in EcN to express α-hemolysin, thereby enabling optogenetic-bacterial combination therapy [107]. Furthermore, *E. coli*@Cu₂O microbial nanohybrids cooperatively induce ferroptosis and cuproptosis under photothermal enhancement, reversing immune suppression and exemplifying a new paradigm for bacterium-mediated, photo-triggered multimodal therapy [108].

#### Ultrasound-responsive nanomaterial integration

Coupling ultrasound-responsive modules with endogenous tumor signals, such as acidic pH, GSH, and ROS, creates a finely tuned “mechanical-chemical” cascade that enhances cytotoxicity while minimizing off-site leakage. A TiO₂@CaP core-shell sonosensitizer dissolves its CaP shell under acidic conditions, releasing Ca^2+^. Subsequently, low-intensity focused ultrasound (LIFU) activates the TiO₂ core to generate ROS, inducing mitochondrial dysfunction and markedly amplifying ICD [109]. The semiconductor polymer/lactate oxidase complex SPNLCu uses sonodynamically produced ROS to cleave its linker, sequentially releasing lactate oxidase (LOx) and Cu^2+^. This cascade of lactate depletion, H₂O₂ generation, and cuproptosis triggers potent ICD in a pancreatic cancer model [110]. Similarly, Cu-doped layered double-hydroxide nanosheets (ZCA NSs) release Cu^2+^ in the GSH-rich environment and, upon ultrasound irradiation, initiate a synergistic sonodynamic therapy (SDT)/cuproptosis assault that effectively suppresses tumor growth [111]. PEG-poly(ω-pentadecalactone-co-N-methyldiethyleneamine-co-3,3′-dithiodipropionate-co-3,3′-thiodipropionate) (PEG-PPMDT) NPs, highly sensitive to low pH and elevated GSH/ROS levels, exploit ultrasound-activated artesunate (ART)-Fe^2+^ chemistry to enhance •OH production, accelerating necrosis and improving therapeutic efficacy [112].

Ultrasound not only serves as an external trigger for nanomaterials but also acts as a precision regulator for therapeutic gene expression and delivery in engineered bacteria–nanomaterial hybrid systems, thus enhancing both penetration depth and immune activation. Researchers have introduced GV biosynthetic genes into EcN and covalently coupled DOX to the bacterial surface, creating a theranostic carrier that integrates ultrasound imaging with chemotherapy. Real-time B-mode guidance allows for site-specific insonation, triggering rapid drug release and synergistic immunotherapy, with tumor inhibition rates exceeding 80% [69]. In another study, a thermosensitive genetic circuit places CTLA-4/PD-L1 dual-antibody genes under the control of a 42 to 45 °C promoter. A brief exposure to focused ultrasound induces sustained intratumoral secretion, significantly extending survival while preserving peripheral tissue safety. Another sonogenetic platform utilizes ultrasound-induced hyperthermia to activate an interferon-γ (IFN-γ) circuit, driving M2-to-M1 macrophage repolarization and enhancing CD8^+^ T cell infiltration (Fig. 7B), thereby enabling deep immune reprogramming [57].

### Modular integration of microenvironment-engineered bacteria–nanomaterial hybrid systems

The TME exhibits distinctive physiological and pathological characteristics, including pronounced hypoxia [113], elevated lactate accumulation [114], acidic pH [115], heightened ROS levels [116], and overexpression of specific enzymes [117]. These factors drive tumor growth and metastasis [118] while providing precisely defined targets for the rational design and optimization of nanomaterials, enabling them to deliver potent, site-specific therapeutic effects within the tumor milieu (Fig. 6C).

#### pH-responsive strategies and design principles

Tumor tissues typically maintain an extracellular pH of approximately 6.5 to 6.9, substantially lower than the physiological pH of 7.4 in normal tissues [119]. This Warburg-driven acidification provides an inherent “acid switch” for smart materials: An ideal system should undergo bond cleavage, phase transition, or shell dissolution at pH ≤ 6.8, thereby releasing drug or ion payloads or amplifying ROS, while remaining inert and nontoxic in neutral blood. An acid-responsive promoter (pCadBA) inserted into *E. coli* triggers bacterial autolysis and anti-PD-1 antibody release specifically at pH ≤ 6.8; a single post-ablation injection in a liver tumor model markedly extends survival [120]. Similarly, Salmonella cloaked with a pH-responsive poly-5-hydroxytryptamine/DNAzyme-MnO₂ nanoshell sheds its coating in the acidic tumor environment, exposing the bacteria and locally activating the DNAzyme. This synergy with ROS-amplified sonodynamic and photodynamic therapies more than doubles tumor suppression [121]. In another approach, an acid-labile CaCO₃ shell encapsulating EcN liposomes remains stable during circulation but dissolves upon reaching the tumor, releasing the liposomal drug cargo, while the bacteria continuously secrete granulocyte-macrophage colony-stimulating factor (GM-CSF), thus establishing a dual-modality of “acid-triggered drug delivery and sustained immune activation” [122].

#### ROS-responsive strategies and design principles

The hypermetabolic state and chronic hypoxia in tumor cells continuously elevate intracellular and extracellular ROS levels. Overactivation of the mitochondrial electron transport chain and NADPH oxidases further exacerbates oxidative stress, providing a natural trigger for ROS-sensitive nanoplatforms. These constructs typically incorporate redox-labile motifs, such as sulfides, selenides, or aryl-boronate esters, which are easily cleaved or oxidized by H₂O₂, O₂•^−^, or •OH. Upon reaching the tumor, bond scission or oxidative conversion rapidly releases therapeutic payloads and regenerates ROS, initiating an “endogenous-trigger-positive-feedback amplification” lethal cascade while remaining inert under normal physiological redox conditions.

Engineered bacteria–nanomaterial hybrid systems can function as intrinsic amplifiers of ROS and immune synergy. Ultrasound-responsive engineered bacteria (UEB), when implanted intratumorally, undergo rapid local heating under low-intensity ultrasound, cleaving an encapsulated catalase-inhibitory peptide. This increases intratumoral O₂ levels, simultaneously boosting ROS to enhance both radiotherapy and sonodynamic effects [123]. Lactobacilli cloaked with ZIF-67 remain viable within hypoxic niches and deliver Co^2+^ cores; the released Co^2+^ catalyzes the conversion of H₂O₂ to ·OH, synergizing with bacteria-induced immune activation to suppress tumor growth [124]. HA-camouflaged engineered *E. coli* trigger high ROS-mediated pyroptosis in the colonic TME while enhancing antigen presentation and memory T cell formation, markedly improving survival in otherwise refractory tumors [125]. Aryl-boronate-ester-containing 4-hydroxymethylphenylboronic acid pinacol ester (HPAP)-docetaxel (DTX)/cinnamaldehyde NPs degrade rapidly in ROS-rich environments, releasing docetaxel. Simultaneously, cinnamaldehyde-induced mitochondrial injury further elevates ROS, achieving dual suppression of tumor growth and metastasis while potentiating anti-PD-1 therapy [126]. In a hypoxic, ROS-abundant glioma model, a PD-L1/ROS dual-targeting temozolomide (TMZ)-loaded nanoplatform accelerates drug release, triggering a ROS surge that induces mitochondrial apoptosis and effectively retards tumor progression [127,128].

Future research should focus on precisely quantifying ROS activation thresholds, conducting safety studies on bacteria–host redox-balance interactions, and developing clinically controllable dosing hardware, thereby laying the foundation for the clinical translation of ROS-mediated, microenvironment-responsive therapies.

#### Enzyme-responsive strategies and design principles

Pathological overexpression of matrix metalloproteinases (MMPs), especially MMP-2 and MMP-9, cathepsin B (Cat B), urokinase-type plasminogen activator (uPA), hyaluronidase, glutathione *S*-transferase (GST), and related enzymes within the TME, provides an intrinsic “molecular ZIP code” for enzyme-cleavable smart nanoplatforms. Unlike pH- or temperature-triggered systems, enzymatic activation occurs under mild conditions with high substrate specificity, enabling highly selective, lesion-targeted therapy [129].

Currently, enzyme-responsive NPs can be anchored to tumor-colonizing engineered bacteria using “carry-and-escort” methods, including bioorthogonal click chemistry, SpyTag-SpyCatcher coupling, electrostatic layer-by-layer assembly, or fusion of liposomes/OMVs. These NPs can also be assembled into detachable “hitchhiking” complexes. Once the bacteria home to the tumor via chemotaxis or hypoxia-driven proliferation, locally overexpressed enzymes cleave the NP shells. For example, lactate dehydrogenase cleaves a tumor necrosis factor-α (TNF-α)-loaded liposomal coat, markedly enhancing α-PD-1 efficacy [130]. Similarly, Cat B activates PD-L1 peptide/DOX dual-loaded NPs, facilitating simultaneous drug release and checkpoint blockade, nearly completely suppressing melanoma growth [131]. Additionally, engineered bacteria–nanomaterial hybrid systems can amplify enzyme-responsive therapy through 3 distinct strategies: bacterial-directed enzyme prodrug therapy (BDEPT), where Salmonella expressing nitroreductase NfsA converts the prodrug CB1954 into a cytotoxic metabolite within the tumor, enabling precise ablation with minimal systemic toxicity [132,133]; the “hitchhiking” approach, where drug-loaded NPs are co-delivered with bacteria, whose secreted enzymes rupture the NP shell and simultaneously activate antitumor immunity [134]; and synthetic genetic circuits that fuse enzyme genes with TME-responsive promoters, allowing collagenase or nitroreductase expression exclusively within the tumor, enabling a sequential “matrix-remodeling then prodrug activation” process [135].

Through this multidimensional integration, enzyme-responsive nanomaterials combined with bacterium-based enzyme modules achieve a 3-tier synergy—matrix degradation, redox disequilibrium, and immune amplification—in multiple solid tumor models. To advance these platforms into clinical applications, future efforts must focus on establishing tunable enzyme-activity thresholds, rigorously evaluating bacteria–host symbiosis safety, and developing real-time, closed-loop imaging systems for dose monitoring.

Overall, the optimization of physically responsive nanoplatforms relies on 3 interconnected pillars: advanced surface engineering, integration of programmable physical-response modules, and TME sensitivity. Precise surface modification extends circulation time, provides immune camouflage, and directs tumor homing, while externally applied light, heat, magnetic fields, or ultrasound facilitates nanostructural disassembly, ROS amplification, and metal-ion release on demand [110]. pH-, ROS-, and enzyme-cleavable linkers add a second layer of control, ensuring hierarchically gated, site-specific drug release. Incorporating engineered bacteria–nanomaterial hybrid systems introduces active chemotaxis and remotely inducible gene expression.

## Challenges and Prospects for Clinical Translation

### Principal barriers and potential solutions

The convergence of engineered bacteria–nanomaterial hybrid systems with physically responsive nanomaterials represents an innovative frontier in precision oncology. While preclinical studies have demonstrated impressive therapeutic outcomes, several critical obstacles must be addressed before these hybrid platforms can be translated into clinical practice. Therapeutic potency must be balanced against controllability. Greater bacterial proliferation may improve colonization but increase dissemination risk; stronger immune stimulation may enhance tumor rejection but aggravate inflammatory toxicity; and more intense physical activation may improve local ablation but compromise bacterial viability or adjacent tissues. Future designs should therefore define a safe and reproducible activity window rather than maximizing each module independently.

#### Safety

Engineered bacteria–nanomaterial hybrid systems offer inherent advantages such as active tumor homing and in situ proliferation. However, their clinical application is constrained by substantial biosafety concerns [12]. Upon administration to humans, these live biotherapeutic products (LBPs) may trigger unpredictable immune responses, off-target dissemination [136], and inflammatory sequelae, with a theoretical risk of virulence reversion [86]. As a result, current strategies prioritize the use of inherently nonpathogenic strains, such as EcN and various *Bifidobacterium* species, which are further detoxified by deleting remaining virulence genes to minimize pathogenicity and immunogenicity. Auxotrophic “suicide” circuits, which eliminate key biosynthetic genes, ensure bacterial survival only in the presence of nutrients absent from host tissues, thereby restricting systemic propagation [136]. Synthetic biology has also led to the development of versatile kill switches; for example, CRISPR-Cas self-destruct circuits can achieve >99% bacterial clearance in animal models [137]. A recently developed “Devil-Angel” bifunctional essential-gene switch maintains low-level expression for normal survival but, upon specific stimuli, triggers high-level expression of lethal genes. Long-term passaging confirmed its stability and reliability [137]. Additionally, the Danino group’s quorum-sensing circuit induces partial bacterial lysis once population density exceeds a threshold, synchronously releasing therapeutic payloads and enabling precise, dynamic control over both bacterial numbers and drug dose [138]. Collectively, these multilayered safety designs support the further development of bacteria-based anticancer platforms, but their clinical translation still requires careful immune-toxicity assessment and controllable clearance strategies [57,139]

Immune safety should also be considered separately from bacterial containment. The same microbial signals that support antitumor adjuvanticity, including LPS, flagellin, CpG DNA, and OMV-associated components, may provoke excessive inflammation, cytokine release, complement activation, or endotoxin-related toxicity if bacterial burden and release kinetics are not tightly controlled. Preexisting antibacterial immunity or recent antibiotic exposure may further alter colonization, clearance, and therapeutic reproducibility. These risks are particularly relevant in immunocompromised patients, for whom dose escalation, route selection, rescue antibiotics, and immune monitoring should be more conservative [12,136]. Clinically, platform termination should use layered control: withdrawal of external stimuli to stop material-mediated activation, predefined rescue antibiotics, and inducible kill switches or auxotrophic containment to clear the bacterial component. Host immune clearance may contribute, but its variability means that monitoring and rescue criteria should be prespecified rather than left to spontaneous elimination.

Physically responsive nanomaterials offer considerable promise for anticancer therapy, but their clinical deployment is still hindered by safety concerns. Material toxicity and immunogenicity are closely linked to particle size, morphology, and surface chemistry. Suboptimal designs can provoke excessive ROS generation, cytotoxicity, and DNA damage, while poorly degradable constructs may accumulate in vivo and cause chronic toxicity [140]. Furthermore, safety data derived from animal models or healthy volunteers often fail to capture the real-world risks faced by cancer patients, particularly those with compromised immunity [122]. Risk mitigation must therefore begin at the design stage: Precise control over particle size, shape, and surface functionalization can reduce off-target uptake and immune activation, while the use of degradable platforms—such as liposomes, biopolymers, or hybrid organic–inorganic matrices—can minimize long-term tissue retention. Simultaneously, a comprehensive toxicity-assessment framework is required, combining advanced in vitro systems, organ-on-a-chip models, and companion diagnostic tools to monitor toxic and immune responses in real time and guide iterative optimization. Implementing these strategies should markedly enhance the safety profile of nanomaterials and accelerate their clinical translation [122].

#### Complexity of the human in vivo environment

Once administered into the human body, these integrated biohybrid platforms must navigate a highly dynamic and unpredictable environment. Following intravenous injection, most bacterial biohybrids are rapidly cleared by the host immune system, with only a small fraction reaching the tumor, where they can proliferate to concentrations of approximately 10^8^ to 10^9^ colony-forming units (CFU) g^−1^ [74]. Tumor tropism is primarily driven by the bacteria’s innate chemotaxis toward the hypoxic, immunosuppressed TME. However, off-target dissemination remains a concern, potentially leading to colonization of healthy tissues and immune-related adverse events [141]. Interpatient heterogeneity complicates matters further, as variations in tumor hypoxia, pH, and immune status can render synthetic genetic circuits ineffective in some individuals [43]. For physically responsive nanomaterials, tumor accumulation predominantly relies on the EPR effect, the magnitude of which varies greatly depending on tumor type, vascular permeability, and stromal architecture. This results in substantial interpatient differences in biodistribution and clearance [142]. Additionally, the depth of penetration achievable by external physical stimuli is limited; for instance, NIR light typically penetrates only a few millimeters of tissue, limiting its effectiveness against deep-seated tumors [99].

Several complementary strategies have been proposed to address these challenges. First, tumor microenvironment-responsive genetic circuits can be engineered to activate therapeutic gene expression only upon sensing specific cues such as hypoxia, acidic pH, or excess lactate [143]. Incorporating multiple microenvironmental signals within the same circuit further enhances specificity and minimizes off-target activation in healthy tissues [138]. In parallel, virulence-attenuated strains equipped with suicide mechanisms can effectively restrict bacterial survival in nontumor compartments [144]. Second, the delivery efficiency of nanomaterials can be enhanced by decorating their surfaces with tumor-specific ligands or by transiently remodeling tumor vasculature and stroma to improve intratumoral accumulation and penetration. Third, the penetration limits of external stimuli can be addressed by using focused ultrasound to trigger heat-sensitive gene switches [145], or by attaching magnetic NPs to bacteria and guiding them toward deep-seated lesions with an external magnetic field [57]. Finally, to maximize synergistic efficacy, spatiotemporal coordination between bacterial activity and nanomaterial release is essential. One practical solution is to construct hybrid systems in which engineered bacteria–nanomaterial hybrid systems are cloaked with or carry drug-loaded NPs, enabling simultaneous delivery and co-release of therapeutics [146]. Animal studies have shown that such hybrids combine the active penetration and immunomodulatory capacity of bacteria with the precise cytotoxicity of nanomaterials, markedly enhancing antitumor efficacy while markedly reducing systemic toxicity [147].

#### Process standardization and quality control

Synergistic platforms that combine engineered bacteria with physically responsive nanomaterials merge living bacteria with functional materials, creating complex manufacturing workflows that are currently poorly standardized—an important bottleneck to clinical translation [86]. Such integrated systems are highly sensitive to fluctuations during culture, loading, and storage; even minor environmental changes can compromise their bioactivity. Similarly, key attributes of nanomaterials—such as particle size, drug loading content, and stimulus responsiveness—tend to vary during scale-up production [138]. When these components are integrated into a single hybrid platform, the complexity increases: Factors like loading ratio, conjugation chemistry, and functional stability must be optimized, yet aligning these variables is challenging, leading to substantial batch-to-batch variability [86].

To overcome these challenges, Good Manufacturing Practice (GMP)-compliant, automated manufacturing workflows should be established. By leveraging best practices from biopharmaceutical production—such as large-scale bacterial fermentation and continuous-flow nanomaterial synthesis—an integrated pipeline encompassing culture, payload loading, purification, and lyophilization can markedly improve product stability and consistency across batches [148]. Product release testing should follow a predefined quality-control (QC) checklist covering bacterial viability, genetic stability, plasmid retention, kill-switch performance, antibiotic sensitivity, nanomaterial loading, particle size distribution, therapeutic-cargo identity and payload leakage, endotoxin level, sterility, stimulus responsiveness or activation-device calibration where applicable, storage stability, and batch-to-batch reproducibility. Propidium monoazide–quantitative polymerase chain reaction (PMA-qPCR), flow cytometry, sequencing-based circuit verification, endotoxin assays, and physicochemical characterization can then be combined to assess both the living and material components [149]. Early and ongoing dialogue with regulatory authorities will facilitate the development of tailored process and quality standards for these complex combination products, ultimately improving product controllability and regulatory feasibility [11].

#### Variability in clinical applicability

To date, most validation of engineered bacteria–nanomaterial hybrid systems has been conducted in small-animal models. However, substantial interspecies differences in immune system composition and TME architecture limit the predictive value of these models for human translation [150]. Therapeutic regimens that appear safe and effective in rodents may present unforeseen risks in the clinic—particularly in immunocompromised patients—such as persistent bacterial colonization or opportunistic infections [150]. Additionally, pronounced interpatient variability in tumor immunological status, stromal organization, and vascular permeability can materially affect bacterial engraftment and nanomedicine accumulation, thereby modulating overall therapeutic outcomes. Heterogeneity in hypoxia, necrosis, vascular abnormality, stromal density, immune exclusion, and local microbial composition may lead to uneven bacterial colonization, variable nanomaterial accumulation and activation, inconsistent immune conversion, and heterogeneous therapeutic output across tumor types or even across different regions of the same lesion. Tumors with hypoxic or necrotic regions, permissive vascular and stromal architecture, immune-excluded features amenable to immune conversion, and lesions accessible to local injection or external activation may be better suited to this strategy, whereas recent antibiotic exposure, altered microbiome status, or severe immunosuppression may reduce colonization predictability or increase safety risk.

To enhance the clinical applicability of these synergistic bacterium–nanomaterial platforms, several measures are advisable. First, more predictive preclinical models—such as humanized mice, patient-derived organoids and xenografts, and organ-on-a-chip systems—should replace conventional rodent assays, as they more accurately replicate the human TME and can help identify potential risks earlier [151]. Based on these data, patient selection algorithms in future trials should integrate molecular imaging with biomarker profiling to ensure that therapy is directed at those most likely to benefit. Future studies should also stratify models and patients by hypoxia, necrosis, stromal barriers, vascular permeability, immune phenotype, antibiotic exposure, and microbiome status to improve cross-study comparability. Administration route should also be matched to disease setting and control requirements: Intratumoral delivery provides high local exposure and easier rescue, intravenous delivery may reach disseminated lesions but increases dissemination risk, oral delivery is more suitable for gastrointestinal or probiotic platforms, intraperitoneal delivery may fit peritoneal disease, and post-ablation delivery may exploit an inflamed residual tumor bed while remaining compatible with local physical triggers. For example, tumor IL-10 receptor expression has been proposed as a biomarker for predicting the efficacy of bacterial therapeutics and could guide personalized treatment protocols [152,153]. Additionally, an integrated “diagnosis-plus-therapy” paradigm—combining real-time imaging with dynamic monitoring—should be implemented in early-phase studies to track the in vivo distribution and behavior of these biohybrid platforms. This would allow for prompt dose adjustments and optimized interventions [152]. Finally, a unified clinical-evaluation framework that incorporates standardized enrollment criteria, outcome measures, and follow-up schedules is crucial for improving cross-study comparability and data robustness, thereby accelerating regulatory approval.

#### Regulatory and ethical barriers

Regulatory and ethical challenges remain substantial for bacterium–nanomaterial combination therapies, which embody live microbial, biologic, and nanoscale components but lack a clearly defined classification or approval pathway. As a composite modality, these therapies face a lack of harmonized technical standards and evaluation criteria across research, manufacturing, and clinical trial stages, severely hindering clinical translation [86]. Long-term safety concerns—such as horizontal gene transfer, dissemination of resistance determinants, and the feasibility of emergency-clearance mechanisms—are still poorly understood, and no comprehensive risk-assessment framework is in place. Ethically, the in-body use of such integrated systems raises concerns regarding off-target colonization, immune hyperactivation, and accidental environmental release; the limited preclinical data available further complicate trial planning and add uncertainty. To navigate these challenges, regulatory agencies could draw from U.S. Food and Drug Administration (FDA) and European Medicines Agency (EMA) precedents by designating such systems as “combination products” or “complex biologics” and issuing technical guidelines covering safety, toxicology, and efficacy testing, with a flexible, product-specific review process [86]. The concurrent deployment of companion diagnostics and real-time monitoring would provide dynamic safety data and enhance risk management. Finally, establishing multidisciplinary review panels—comprising experts from bioengineering, clinical medicine, ethics, and regulatory science—would improve the scientific rigor, transparency, and public acceptance of risk assessments.

### Current landscape of clinical research and translation

Current clinical data mainly come from bacteria-based therapeutics, bacterial derivatives, and physical or nanomaterial activation modules. By contrast, fully integrated live bacteria–nanomaterial hybrid systems remain largely preclinical. This section distinguishes clinically tested bacterial therapeutics, clinically explored nanomaterial or physical-activation technologies, bacterial derivatives such as OMVs, and true integrated live bacteria–nanomaterial hybrid systems.

**Fig. 8.**
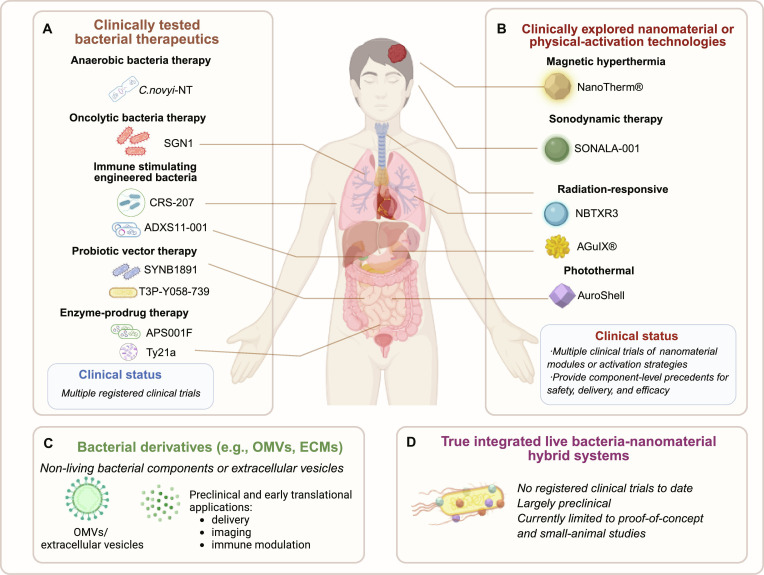
Clinical and translational landscape of bacteria-based therapeutics, bacterial derivatives, nanomaterial activation modules, and integrated bacteria–nanomaterial hybrid platforms. (A) Clinically tested bacterial therapeutics. (B) Clinically explored nanomaterial and physical-activation technologies. (C) Preclinical bacterial derivatives, including OMVs and ECMs. (D) Preclinical integrated live bacteria–nanomaterial hybrid systems.

To avoid conflating different evidence levels, translational examples in this section are further classified as in vitro proof-of-concept, small-animal efficacy, large-animal safety or biodistribution, component-level clinical precedent, bacterial derivatives in translational development, or true integrated live hybrid systems, depending on the available evidence (Fig. 8).

#### Clinical advances of physically responsive nanomaterials in oncology

In recent years, tumor therapy strategies utilizing physically responsive nanomaterials have advanced into clinical trials, where they have begun to demonstrate favorable safety profiles and translational potential (Table 4). In PTT, Nanospectra Biosciences’ gold-silica nanoshells (AuroShell) generate highly localized heat when exposed to NIR laser light. In a prostate cancer study (AuroLase, NCT04240639) involving approximately 100 patients, most tumors were completely ablated with no clinically meaningful recurrences or serious adverse events reported [151]. The platform is also being explored in a trial for metastatic lung cancer (NCT01679470), further expanding its clinical scope [154]. In magnetothermal therapy, MagForce’s NanoTherm system delivers superparamagnetic iron-oxide NPs into the tumor or resection cavity of glioblastoma, followed by exposure to an alternating magnetic field to generate local hyperthermia in combination with radiotherapy. In a single-arm phase II study of 59 patients with recurrent glioblastoma treated with intratumoral NanoTherm and reduced-dose external beam radiotherapy, median overall survival from first recurrence reached 13.4 months with an acceptable safety profile [155]. NanoTherm received a CE mark in Europe in 2010 as a medical device for the treatment of brain tumors, and an ongoing adjuvant trial (NCT06271421) is currently evaluating its impact on survival in a larger recurrent glioblastoma cohort [128]. Although a prostate cancer trial (NCT05010759) was halted early due to recruitment challenges [156], the favorable results in brain tumors highlight the platform’s potential for other solid malignancies. SDT is progressing rapidly. SonALAsense’s 5-aminolevulinic-acid-based nanosensitizer, SONALA-001 (NCT05370508), generates cytotoxic ROS when activated by focused ultrasound. A phase I study in recurrent glioblastoma established a safe acoustic dose [157], and a subsequent phase II trial in pediatric DIPG (NCT05123534) showed tumor-growth suppression in several patients without serious adverse events, highlighting the platform’s promise for treating deep-seated brain tumors [158]. Nanobiotix’s hafnium-oxide radiosensitizer, NBTXR3, amplifies radiotherapy and succeeded in a phase III soft tissue sarcoma trial, earning EU marketing approval in 2019. It is now being tested in several other solid tumors, including non-small cell lung cancer (NCT04505267), esophageal cancer (NCT04615013), oropharyngeal squamous carcinoma (NCT01946867), and pancreatic cancer (NCT04484909). AGuIX, another NP radiosensitizer, is also advancing clinically: A phase I/II study combining it with radiotherapy and temozolomide for newly diagnosed glioblastoma is underway (NCT04881032); a parallel phase I/II trial pairing it with magnetic resonance imaging (MRI)-guided stereotactic body radiotherapy (SBRT) for pancreatic and lung cancers is ongoing (NCT04789486); and a phase I dose-finding study in multiple brain metastases (NCT02820454) has established the recommended dose, with a phase II efficacy trial in progress (NCT03818386). Collectively, these studies provide component-level clinical precedents for physical-activation modules, but they should not be interpreted as clinical validation of complete live bacteria–nanomaterial hybrid systems.

**Table 4.** Clinical trials of physically responsive nanomaterials against tumors: Component-level clinical precedents for activation modules. Information from ClinicalTrials.gov. The stage of a clinical trial studying a drug or biological product is based on FDA definitions. The phase is based on the study’s objective, the number of participants, and other characteristics. There are 5 phases: early phase 1 (formerly listed as phase 0), phase I, phase II, phase III, and phase IV. Not applicable is used to describe trials without FDA-defined phases, including trials of devices or behavioral interventions.

Nanomaterial	Therapy	Pathology	ClinicalTrials ID	Study title	Phase	Date	Status
NanoTherm	Magnetic hyperthermia	Prostate cancer	NCT05010759	Study of focal ablation of the prostate with NanoTherm therapy system for intermediate-risk prostate cancer	Not applicable	08/2021	Terminated
		Glioblastoma (GBM)	NCT06271421	NanoTherm in adjuvant therapy of glioblastoma multiforme	Not applicable	02/2024	Recruiting
AGuIX	Radiotherapy	Glioblastoma	NCT04881032	AGuIX nanoparticles with radiotherapy plus concomitant temozolomide in the treatment of newly diagnosed glioblastoma	Phase I/II	03/2022	Active, not recruiting
		Brain metastases	NCT03818386	Radiotherapy of multiple brain metastases using AGuIX	Phase II	03/2019	Active, not recruiting
		Brain metastases	NCT04899908	Stereotactic brain-directed radiation with or without Aguix gadolinium-based nanoparticles in brain metastases	Phase II	09/2021	Recruiting
		Lung and pancreatic	NCT04789486	Nano-SMART: nanoparticles with MR guided SBRT in centrally located lung tumors and pancreatic cancer	Phase I/II	05/2021	Recruiting
		Gynecologic cancer	NCT03308604	AGuIX gadolinium-based nanoparticles in combination with chemoradiation and brachytherapy	Phase I	05/2018	Unknown
		Brain metastases	NCT02820454	Radiosensitization of multiple brain metastases using AGuIX gadolinium based nanoparticles	Phase I	06/2016-06/2019	Completed
AuroShell	Photothermal therapy	Prostate cancer	NCT04240639	An extension study MRI/US fusion imaging and biopsy in combination with nanoparticle directed focal therapy for ablation of prostate tissue	Not applicable	01/2020	Active, not recruiting
		Head and neck	NCT00848042	Pilot study of AuroLase(tm) therapy in refractory and/or recurrent tumors of the head and neck	Not applicable	02/2009-02/2017	Completed
		Prostate cancer	NCT02680535	MRI/US fusion imaging and biopsy in combination with nanoparticle directed focal therapy for ablation of prostate tissue	Not applicable	02/2016-03/2021	Completed
AuroLase	Photothermal therapy	Lung cancer	NCT01679470	Efficacy study of AuroLase therapy in subjects with primary and/or metastatic lung tumors	Not applicable	09/2012-11/2016	Terminated
NBTXR3	Radiotherapy	NSCLC	NCT04505267	NBTXR3 and radiation therapy for the treatment of inoperable recurrent non-small cell lung cancer	Phase I	02/2021	Recruiting
		Head and neck	NCT04862455	NBTXR3, radiation therapy, and pembrolizumab for the treatment of recurrent or metastatic head and neck squamous cell cancer	Phase II	04/2021	Recruiting
		Head and neck	NCT04892173	JNJ-90301900 (NBTXR3) activated by radiotherapy with or without cetuximab in LA-HNSCC	Phase III	01/2022	Recruiting
		Pancreatic cancer	NCT04484909	NBTXR3 activated by radiation therapy for the treatment of locally advanced or borderline-resectable pancreatic cancer	Phase I	07/2020	Recruiting
		Esophageal cancer	NCT04615013	NBTXR3, chemotherapy, and radiation therapy for the treatment of esophageal cancer	Phase I	11/2020	Recruiting
		Sarcoma	NCT01433068	NBTXR3 crystalline nanoparticles and radiation therapy in treating patients with soft tissue sarcoma of the extremity	Phase I	09/2011-10/2020	Completed
		Sarcoma	NCT02379845	NBTXR3 crystalline nanoparticles and radiation therapy in treating randomized patients in two arms with soft tissue sarcoma of the extremity and trunk wall	Phase II/III	03/2015-04/2021	Completed
		Squamous cell carcinoma of the oropharynx	NCT01946867	NBTXR3 and radiation therapy in treating patients with locally advanced SCC of the oral cavity or oropharynx	Phase I	09/2013-12/2022	Unknown
SPIO	Magnetic hyperthermia	Prostate cancer	NCT02033447	Magnetic nanoparticle thermoablation-retention and maintenance in the prostate: A phase 0 study in men	Phase I	01/2014-05/2017	Completed
ThermoDox	High-intensity focused ultrasound	Pediatric cancer solid tumors	NCT02536183	A phase I study of lyso-thermosensitive liposomal doxorubicin and MR-HIFU for pediatric refractory solid tumors	Phase I	08/2015-08/2023	Terminated
	Radiofrequency ablation	Colon cancer	NCT01464593	Phase II study of ThermoDox as adjuvant therapy with thermal ablation (RFA) in treatment of metastatic colorectal cancer (mCRC)	Phase II	02/2017-09/2022	Terminated
		Hepatocellular carcinoma	NCT02112656	Study of ThermoDox with standardized radiofrequency ablation (RFA) for treatment of hepatocellular carcinoma (HCC)	Phase III	04/2014-07/2024	Completed
	Microwave hyperthermia	Breast cancer	NCT00826085	Phase I/II study of ThermoDox with approved hyperthermia in treatment of breast cancer recurrence at the chest wall	Phase II	01/2009-01/2017	Completed
	MRGFUS	Breast cancer	NCT03749850	Image-guided targeted doxorubicin delivery with hyperthermia to optimize loco-regional control in breast cancer	Phase I	11/2018-02/2021	Unknown
		Liver cancer	NCT02181075	Targeted chemotherapy using focused ultrasound for liver tumors	Phase I	07/2014-11/2018	Completed
SONALA-001	MRGFUS	GBM	NCT05370508	A study of sonodynamic therapy using SONALA-001 and Exablate 4000 type 2.0 in subjects with recurrent GBM	Phase II	05/2022-07/2024	Terminated
		Diffuse intrinsic pontine glioma	NCT05123534	A phase II study of sonodynamic therapy using SONALA-001 and Exablate 4000 type 2.0 in patients with DIPG	Phase II	11/2021-09/2024	Suspended

GBM, glioblastoma; NSCLC, non-small cell lung cancer; LA-HNSCC, locally advanced head and neck squamous cell carcinoma; DIPG, diffuse intrinsic pontine glioma; SCC, squamous cell carcinoma; mCRC, metastatic colorectal cancer; MR, magnetic resonance; MRI, magnetic resonance imaging; US, ultrasound; RFA, radiofrequency ablation; SBRT, stereotactic body radiotherapy; MR-HIFU, magnetic resonance-guided high-intensity focused ultrasound; MRGFUS, magnetic resonance-guided focused ultrasound; SPIO, superparamagnetic iron oxide

#### Clinical progress of bacteria-based therapeutics and related hybrid strategies

Most clinical examples discussed in this section represent bacteria-based therapeutics or related component technologies rather than fully integrated live bacteria–nanomaterial hybrids (Table 5). In the realm of anaerobic bacterial therapy, intratumoral injection of attenuated *Clostridium novyi*-NT (nontoxic) spores induced robust local necrosis and systemic immune activation in phase I/Ib studies of advanced solid tumors (NCT01924689/NCT03435952); a parallel phase Ib trial is evaluating synergy with pembrolizumab [43]. In lytic-bacterial approaches, the attenuated *S. Typhimurium* strain SGN1, engineered to secrete l-methioninase and starve tumors of an essential amino acid, is currently being tested in a multinational phase I/IIa study across 23 sites (NCT05038150/NCT05103345), where complete and partial responses have been documented, highlighting its broad-spectrum potential. Immunostimulatory vectors based on *Listeria monocytogenes* are also advancing: CRS-207, which expresses the mesothelin antigen, has shown acceptable tolerability and initial efficacy in pancreatic cancer and malignant pleural mesothelioma trials (NCT05014776/NCT03175172) [150,159,160], while *Axalimogene filolisbac* (ADXS11-001), targeting HPV16-E7, induced immune activation and clinical benefit in a cervical cancer phase II study (NCT02164461) and prolonged progression-free survival to 68 weeks in anal cancer patients (NCT02399813) [161]. Probiotic carriers are also gaining momentum: *SYNB1891*, derived from EcN and designed to release cyclic dinucleotides that activate STING under hypoxia, demonstrated a favorable safety profile and on-target immune activation in a phase I trial (NCT04167137), with phase II now underway [162]. *Yersinia*-based *T3P-Y058-739*, developed by T3 Pharmaceuticals to deliver therapeutic proteins via the type III secretion system, is undergoing first-in-human phase I/II testing (NCT05120596) alongside exploration of PD-1 blockade combinations, and has been acquired by Boehringer Ingelheim for further development. In enzyme-prodrug therapies, Anaeropharma’s APS001F (*Bifidobacterium longum* expressing cytosine deaminase) safely converted 5-fluorocytosine (5-FC) to 5-FU intratumorally in a phase I trial (NCT01562626) [48], while its upgraded IL-12-armed variant, bacTRL-IL12, though halted early (NCT04025307), validated the tumor-targeted suicide-enzyme concept. Finally, oral Ty21a typhoid-vaccine bacteria are being repurposed for non-muscle-invasive bladder cancer (NCT03421236), inducing measurable immune responses and offering a potential alternative to BCG. These studies provide useful clinical precedents for individual modules, but further evidence is required before complete live bacteria–nanomaterial hybrid platforms can be considered clinically validated.

**Table 5.** Clinical trials of engineered bacteria against tumors: Component-level clinical precedents for bacterial therapeutics. Data were obtained from ClinicalTrials.gov.

Species	Pathology	ClinicalTrials ID	Phase	Study period	Status
*VNP20009*	Advanced solid tumors	NCT00006254	Phase I	09/2003-12/2007	Completed
	Solid tumors	NCT00004216	Phase I	02/2004-12/2007	Completed
	Neoplasms	NCT00004988	Phase I	03/2002-10/2002	Completed
*Salmonella*	Multiple myeloma	NCT03762291	Phase I	12/2018-02/2025	Completed
*S. Typhimurium SGN1*	Advanced solid tumors	NCT05038150	Phase I	09/2021	Recruiting
	Advanced solid tumors	NCT05103345	Phase I	11/2021-08/2024	Recruiting
*S. Typhimurium Saltikva*	Pancreatic cancer	NCT04589234	Phase II	10/2020	Active, not recruiting
BCG	Small cell lung cancer	NCT00006352	Phase III	07/2003-07/2012	Completed
BCG*: S1602*	Bladder cancer	NCT03091660	Phase III	03/2017	Active, not recruiting
*Clostridium novyi-NT Spores*	Solid tumors	NCT01924689	Phase I	08/2013-08/2019	Completed
Solid tumors	NCT03435952	Phase I	02/2018	Active, not recruiting
*Listeria: CRS-207*	Pancreatic cancer	NCT01417000	Phase II	08/2011-04/2018	Completed
	Pancreatic cancer	NCT05014776	Phase II	08/2021	Active, not recruiting
	MPM	NCT03175172	Phase II	06/2017-03/2019	Terminated
*Listeria monocytogenes*	Cervical cancer	NCT02853604	Phase III	08/2016-02/2023	Terminated
*Akkermansia muciniphila*	NSCLC or RCC	NCT05865730	Phase II	03/2023	Recruiting
*Gut microbiome*	Breast and lung cancer	NCT04857697	Phase I	04/2021-04/2023	Completed
*Clostridium novyi-NT*	Solid tumors	NCT00358397	Phase I	07/2006-10/2016	Terminated
*Bifidobacterium longum*	Solid tumors	NCT04025307	Phase I	07/2019-03/2021	Terminated
*E. coli SYNB1891*	Solid tumors and lymphoma	NCT04167137	Phase I	11/2019-11/2023	Terminated
*ADXS-503*	Lung cancer	NCT03847519	Phase II	02/2019-05/2024	Completed
*Axalimogene filolisbac (ADXS11-001)*	Carcinoma of the cervix	NCT02164461	Phase I	06/2014-12/2023	Completed
	Metastatic squamous cell carcinoma	NCT02399813	Phase II	03/2015-03/2023	Completed
*T3P-Y058-739 (T3P)*	Solid tumors	NCT05120596	Phase I/II	11/2021	Recruiting
*Ty21a (Vivotif)*	Bladder cancer	NCT03421236	Phase I	02/2018-04/2021	Unknown

MPM, malignant pleural mesothelioma; NSCLC, non-small cell lung cancer; RCC, renal cell carcinoma

#### Emerging trends in engineered bacteria–nanomaterial hybrid systems

To date, no registered clinical trials have combined engineered bacteria with photothermal, magnetothermal, or sonodynamic nanomaterials for cancer therapy; these integrated strategies remain confined to the preclinical stage. Accordingly, these examples should be interpreted as proof-of-concept or small-animal efficacy evidence rather than clinical validation. Nevertheless, selected animal studies illustrate how such integrated designs may work mechanistically. For example, *E. coli* carrying a photothermal agent and a heat-inducible genetic circuit enabled NIR-triggered local heating together with bacterial expression of cytolytic ClyA [103]. This example supports the feasibility of spatially coordinated bacterial programming and material activation, but it remains preclinical and should be interpreted as mechanistic proof-of-concept rather than evidence of clinical readiness.

### Future directions and research roadmap for engineered bacteria–nanomaterial hybrid systems

The synergistic combination of engineered bacteria–nanomaterial hybrid systems represents an emerging therapeutic paradigm with translational potential. Although substantial challenges remain, ongoing advances in synthetic biology, nanotechnology, and interdisciplinary research are steadily clarifying both the scientific trajectory and translational pathway of this field. Rapid progress in synthetic biology is equipping engineered bacteria with highly programmable “smart” capabilities. Precisely designed gene circuits now enable these microbes to sense and respond to TME cues—such as hypoxia, acidosis, and characteristic metabolites—boosting tumor-selective expression of therapeutic payloads and enhancing overall efficacy [163]. Moving forward, modular design principles will be crucial to optimizing genetic stability, environmental robustness, and functional module integration, ensuring durable control over both therapeutic benefits and safety. Simultaneously, the development of physically responsive nanomaterials is advancing, with a focus on improving tissue penetration and expanding multimodal theranostic functionality. Since conventional photothermal agents are limited by shallow light penetration, magnetothermal and sonodynamic platforms have gained prominence. Magnetically responsive nanomaterials can be directed to deep-seated tumors under an external magnetic field, generating local hyperthermia for targeted ablation [164]. Ultrasound-responsive constructs leverage focused ultrasound to generate ROS and cavitation effects, offering noninvasive, potent cytotoxicity against deep tumors [165]. Multimodal systems are also emerging: Nanomicelles responsive to both magnetic fields and acidic pH enable precise tumor localization and efficient drug release, improving therapeutic outcomes in preclinical models [166]. Additionally, enzyme-triggered in situ self-assembly strategies dramatically improve intratumoral accumulation and efficacy [167]. Synergies between engineered bacteria–nanomaterial hybrid systems and other frontier technologies—such as immunotherapy, metabolic modulation, and gene editing—are broadening the therapeutic landscape. Recent studies on controllable living therapeutics, ultrasound-visible or ultrasound-controllable engineered bacteria, and OMV-based tumor vaccines further extend this roadmap. These directions emphasize image-guided localization, externally regulated therapeutic output, and nonreplicating bacterial derivatives, which may help improve controllability while reducing risks associated with replicating live bacteria [4,6,69]. As in situ “living vaccine” carriers, engineered bacteria can deliver tumor-specific antigens and robustly stimulate host antitumor immunity [168]. They can also attenuate immunosuppressive pathways, such as indoleamine 2,3-dioxygenase (IDO), to enhance immune checkpoint blockade [169]. Concurrently, embedding CRISPR-Cas modules in bacteria or nanomaterials enables precise editing of oncogenic pathways, creating new avenues for personalized intervention [86]. Moving forward, research will increasingly focus on tailoring combination platforms to the molecular features of individual tumors, constructing highly personalized bacterium–nanomaterial regimens to maximize therapeutic precision and clinical benefit.

Clinical translation will require coordinated optimization of design, monitoring, and regulation. Artificial intelligence (AI)-assisted modeling may help prioritize material parameters, predict biodistribution, and support patient stratification, but these tools require prospective validation. Companion diagnostics and real-time imaging should be integrated with safety monitoring to track bacterial distribution, nanomaterial activation, immune response, and clearance. Regulatory frameworks should therefore define release specifications, monitoring endpoints, and rescue procedures for these live-material combination products.

## Summary and Outlook

Engineered bacteria–nanomaterial hybrid systems form a dual-active paradigm for precision oncology. The bacterial chassis provides intrinsic tumor tropism, deep tissue penetration, and in situ proliferation, effectively transporting drug or gene cargo into hypoxic, necrotic tumor cores. Simultaneously, programmable nanomaterials, embedded for photothermal, photodynamic, or sonodynamic actuation, can be triggered by external fields to deliver targeted attacks, induce ICD, rapidly expose tumor antigens, and activate the dendritic cell–T cell axis. This approach bridges local tumor debulking with systemic immune memory. Synthetic biology enhances bacteria with finely tuned environmental sensors and fail-safe kill switches, while smart nanoplatforms enable spatiotemporal control over payload release and real-time imaging. Together, these features support more selective and monitorable therapeutic designs, although their clinical benefit remains to be established.

In preclinical studies, prototype platforms have induced antitumor immunity in several solid-tumor models. However, current clinical evidence mainly comes from individual components, including bacterial therapeutics, bacterial derivatives, and nanomaterial or physical-activation modules, rather than from fully integrated live bacteria–nanomaterial hybrids. Before these complete platforms can be considered clinically validated, key challenges in biosafety, immune toxicity, controllable clearance, manufacturing consistency, and long-term efficacy must be addressed. Future studies should therefore combine rigorous preclinical modeling with carefully stratified early-phase trials to define where these systems can provide clear therapeutic benefit.

## Data Availability

Data sharing not applicable to this article as no datasets were generated or analyzed during the current study.
